# Improving Risk Stratification for Patients With Type 2 Myocardial Infarction

**DOI:** 10.1016/j.jacc.2022.10.025

**Published:** 2023-01-17

**Authors:** Caelan Taggart, Karla Monterrubio-Gómez, Andreas Roos, Jasper Boeddinghaus, Dorien M. Kimenai, Erik Kadesjo, Anda Bularga, Ryan Wereski, Amy Ferry, Matthew Lowry, Atul Anand, Kuan Ken Lee, Dimitrios Doudesis, Ioanna Manolopoulou, Thomas Nestelberger, Luca Koechlin, Pedro Lopez-Ayala, Christian Mueller, Nicholas L. Mills, Catalina A. Vallejos, Andrew R. Chapman

**Affiliations:** a BHF Centre for Cardiovascular Science, University of Edinburgh, Edinburgh, United Kingdom; b MRC Human Genetics Unit, Institute of Genetics and Cancer, University of Edinburgh, Edinburgh, United Kingdom; c Department of Medicine, Clinical Epidemiology Division, Karolinska Institute, Stockholm, Sweden; d Department of Emergency and Reparative Medicine, Karolinska University Hospital, Stockholm, Sweden; e Department of Medicine, Karolinska Institute, Stockholm, Sweden; f Usher Institute, University of Edinburgh, Edinburgh, United Kingdom; g Department of Statistical Sciences, University College London, London, United Kingdom; h Cardiovascular Research Institute Basel (CRIB), University Hospital Basel, Basel, Switzerland; i The Alan Turing Institute, London, United Kingdom

**Keywords:** risk prediction, type 2 myocardial infarction, AUC, area under the receiving-operating characteristic curve, GRACE, Global Registry of Acute Coronary Events, hs-cTnI, high-sensitivity cardiac troponin I, hs-cTnT, high-sensitivity cardiac troponin T, ICD-10, International Classification of Diseases-10th Revision, TIMI, Thrombolysis In Myocardial Infarction

## Abstract

**Background:**

Despite poor cardiovascular outcomes, there are no dedicated, validated risk stratification tools to guide investigation or treatment in type 2 myocardial infarction.

**Objectives:**

The goal of this study was to derive and validate a risk stratification tool for the prediction of death or future myocardial infarction in patients with type 2 myocardial infarction.

**Methods:**

The *T2-risk* score was developed in a prospective multicenter cohort of consecutive patients with type 2 myocardial infarction*.* Cox proportional hazards models were constructed for the primary outcome of myocardial infarction or death at 1 year using variables selected a priori based on clinical importance. Discrimination was assessed by area under the receiving-operating characteristic curve (AUC). Calibration was investigated graphically. The tool was validated in a single-center cohort of consecutive patients and in a multicenter cohort study from sites across Europe.

**Results:**

There were 1,121, 250, and 253 patients in the derivation, single-center, and multicenter validation cohorts, with the primary outcome occurring in 27% (297 of 1,121), 26% (66 of 250), and 14% (35 of 253) of patients, respectively. The *T2-risk* score incorporating age, ischemic heart disease, heart failure, diabetes mellitus, myocardial ischemia on electrocardiogram, heart rate, anemia, estimated glomerular filtration rate, and maximal cardiac troponin concentration had good discrimination (AUC: 0.76; 95% CI: 0.73-0.79) for the primary outcome and was well calibrated. Discrimination was similar in the consecutive patient (AUC: 0.83; 95% CI: 0.77-0.88) and multicenter (AUC: 0.74; 95% CI: 0.64-0.83) cohorts. *T2-risk* provided improved discrimination over the Global Registry of Acute Coronary Events 2.0 risk score in all cohorts.

**Conclusions:**

The *T2-risk* score performed well in different health care settings and could help clinicians to prognosticate, as well as target investigation and preventative therapies more effectively. (High-Sensitivity Troponin in the Evaluation of Patients With Suspected Acute Coronary Syndrome [High-STEACS]; NCT01852123)

The universal definition of myocardial infarction differentiates type 1 myocardial infarction due to atherosclerotic plaque rupture and intracoronary thrombosis from type 2 myocardial infarction secondary to oxygen supply and demand imbalance, without atherosclerotic plaque rupture and thrombosis, typically in the setting of another acute systemic or cardiovascular illness.^1^, ^2^, ^3^ Outcomes in patients with type 2 myocardial infarction are poor, with more than two-thirds of patients dead at 5 years.^4^^,^^5^ Although it is understood that noncardiovascular death is prevalent in this population, who are often older and have a higher burden of comorbidity, recent studies suggest that the absolute rates of cardiovascular events are similar to those with type 1 myocardial infarction.^6^, ^7^, ^8^

Early risk stratification is important to inform prognosis and to guide management in patients with acute coronary syndrome. The GRACE (Global Registry of Acute Coronary Events) 2.0 score^9^^,^^10^ and the TIMI (Thrombolysis In Myocardial Infarction) score^11^ are recommended in international guidelines and used widely in clinical practice.^12^, ^13^, ^14^ However, these scores were developed before the introduction of high-sensitivity cardiac troponin assays and the classification of myocardial infarction according to mechanism. Type 2 myocardial infarction is a more heterogeneous condition than type 1 myocardial infarction. Few risk stratification tools have been optimized for this patient group, and none are recommended by current guidelines.

We aimed to derive and validate a new risk stratification tool for use in patients with type 2 myocardial infarction to determine the likelihood of future myocardial infarction or death that could assist clinicians in the targeting of further investigation and secondary prevention.

## Methods

### Derivation cohort

High-STEACS (High-Sensitivity Troponin in the Evaluation of Patients With Suspected Acute Coronary Syndrome) was a stepped-wedge, cluster randomized controlled trial that evaluated the implementation of a high-sensitivity cardiac troponin I (hs-cTnI) assay in consecutive patients with suspected acute coronary syndrome across 10 secondary and tertiary care hospitals in Scotland (NCT01852123). All patients presenting between June 10, 2013, and March 3, 2016, were screened by the attending clinician and prospectively included if cardiac troponin assessment was requested for suspected acute coronary syndrome***.*** Individual patient consent was not sought. The study design has been described in detail previously^3^^,^^6^^,^^15^ and was conducted in accordance with the Declaration of Helsinki with the approval of the Scotland Research Ethics Committee, the Public Benefit and Privacy Panel for Health and Social Care, and by each National Health Service Health Board.

### Validation cohort: single-center, consecutive patients

Consecutive patients >25 years of age presenting to the emergency department at the Karolinska University Hospital in Stockholm with a principal symptom of chest pain and at least one cardiac troponin measurement were enrolled between January 1, 2011, and October 7, 2014.^16^^,^^17^ The hospital’s administrative database was used to identify eligible patients. Patients were excluded if they had an estimated glomerular filtration rate <15 mL/min/1.73 m^2^ or if they presented with ST-segment elevation on the electrocardiogram.

Administrative and laboratory data were linked by the Swedish National Board of Health and Welfare to information on comorbidities and hospital admissions, medications, and deaths from the National Patient Register, the Prescribed Drug Register, and the Cause-of-Death register, respectively.

### Validation cohort: multicenter, international

APACE (Advantageous Predictors of Acute Coronary Syndromes Evaluation) is a prospective, multicenter, international study that enrolled patients from April 25, 2006, until February 2, 2018, with chest pain from 12 centers across 5 countries in Europe (NCT00470587). Adults presenting to the emergency department with chest pain at rest or on minor exertion within 12 hours from presentation were enrolled following receipt of written informed consent. Patients with end-stage kidney failure requiring regular dialysis or with cardiogenic shock were excluded. The study was conducted according to the principles of the Declaration of Helsinki and approved by the local ethics committees.

### Cardiac troponin testing

In the derivation cohort, cardiac troponin levels were measured by using the Abbott ARCHITECT STAT hs-cTnI assay (Abbott Diagnostics). This assay has a limit of detection of 1.2 ng/L, an interassay coefficient of variation of <10% at 4.7 ng/L, and a 99th centile of 16 ng/L in women and 34 ng/L in men.^18^ In both validation cohorts, cardiac troponin levels were measured by using a high-sensitivity cardiac troponin T (hs-cTnT) assay (Roche Elecsys, Roche Diagnostics). This assay has a limit of detection of 5 ng/L, a coefficient of variation of <10% at 13 ng/L, and a uniform 99th centile cutoff point of 14 ng/L.^19^

### Diagnostic adjudication

In all cohorts, all diagnoses were adjudicated in patients with evidence of myocardial injury in accordance with the fourth universal definition of myocardial infarction.^1^ In the derivation cohort, myocardial injury was defined as any hs-cTnI concentration above the sex-specific 99th centile. In the single-center, consecutive patient cohort and the multicenter, international validation cohort, myocardial injury was defined as any hs-cTnT concentration above the uniform 99th centile. In patients with acute myocardial injury, defined as a rise and or fall in cardiac troponin level on serial testing (derivation cohort), a relative change of ≥3 ng/L (single-center consecutive patient cohort), or a predefined absolute change in cardiac troponin concentration (multicenter validation cohort), the diagnosis was adjudicated in accordance with the universal definition.^17^^,^^20^

In all cohorts, 2 physicians independently reviewed all clinical information, with discordant diagnoses resolved by a third reviewer or by consensus as previously described.^6^^,^^17^^,^^21^ In brief, type 1 myocardial infarction was diagnosed in patients with evidence of myocardial injury and symptoms of myocardial ischemia or signs of myocardial ischemia on the electrocardiogram. Patients with symptoms or signs of myocardial ischemia, in which myocardial injury occurred because of increased myocardial oxygen demand or decreased supply secondary to an alternative condition without evidence of atherothrombosis, were diagnosed as type 2 myocardial infarction. Patients with elevated cardiac troponin concentrations without symptoms or signs of myocardial ischemia were classified as having nonischemic myocardial injury (Supplemental Methods).

### Primary and secondary outcomes

The primary outcome was subsequent myocardial infarction or death from any cause at 1 year. The secondary outcome was subsequent myocardial infarction or cardiovascular death at 1 year. In the derivation cohort, regional and national registries were used to ensure complete follow-up for the trial population as previously described.^3^ All outcome events were adjudicated by a panel who were blinded to the index diagnosis and study phase, and subsequent events classified as type 1 or type 4b myocardial infarction included. In the single-center validation cohort, the Swedish National Patient Register was used to retrieve diagnoses for all outcomes. Myocardial infarction was defined by using the International Classification of Diseases-10th Revision (ICD-10) codes I21 or I22. Cardiovascular death was defined by using ICD-10 codes I10 to I15, I20 to I25, I44 to I51, I61, I62.0, I62.9, I63.0 to I63.5, I63.8, I63.9, I64 to I67, and I70 to I73 (Supplemental Table 1). All other deaths were classified as noncardiovascular. In the multicenter, international validation cohort, the cause of death during follow-up was obtained from the patient’s hospital notes, the family physician’s records, and the national registry on mortality. Cardiovascular death was defined as death from myocardial infarction, stroke, heart failure, or sudden cardiac death, or death within 7 days of cardiovascular intervention.

All index admission myocardial infarction events were excluded in all 3 cohorts.

### Statistical analysis

Baseline characteristics were summarized for patients with type 2 myocardial infarction enrolled in the derivation and validation cohorts. Continuous variables are described by using mean ± SD or median (IQR) as appropriate; categorical variables are described as frequencies and percentages. Where data were missing in the derivation cohort, this was assumed to be at random after visual inspection, and multiple imputation using chained equations was performed (Supplemental Figure 1).

Survival analysis was performed by using Cox proportional hazards models for the primary and secondary outcomes separately. For the secondary outcome, noncardiovascular deaths were censored to account for competing risks in a cause-specific model. The derivation of the *T2-risk* score was based on the model constructed for the primary outcome. For both models, covariates were defined a priori based on clinical knowledge and the availability of data, including variables that are objective and readily available to clinicians.^8^^,^^22^ We adopted a parsimonious approach and took into consideration the contribution of variables to model discrimination and calibration. The initial covariate set was defined a priori based on clinical knowledge and the availability of data, including those variables that are objective and readily available to clinicians. Variables for inclusion in the final model were refined considering their univariate association with the primary outcome, their correlation with other covariates, and their association with improvement in discriminatory performance.

Continuous variables, including age, estimated glomerular filtration rate, and heart rate, were modeled by using restricted cubic splines to account for nonlinearity using 4, 3, and 3 knots, respectively. Cardiac troponin concentration was log transformed. Categorical variables included anemia (defined as a hemoglobin concentration <130 g/L in men and <120 g/L in women according to the World Health Organization criteria^23^), ischemic heart disease, diabetes mellitus, previous heart failure hospitalization, and the presence of ischemia on the electrocardiogram. Two strong outliers were identified (based on deviance residuals and DFBeta values) and removed from the analysis. Discrimination was assessed by quantifying the area under the receiving-operating characteristic curve (AUC) and with a bootstrapped C-statistic. Calibration was assessed graphically by plotting observed and predicted risk for each decile of risk in the derivation and validation cohorts. This was also assessed internally by obtaining bias-corrected estimates of predicted vs observed values using a flexible hazard regression model.^24^, ^25^, ^26^ We compared the discriminative performance of *T2-risk* score vs the GRACE 2.0 algorithm using the DeLong test.^10^

Although the *T2-risk* score was derived by using hs-cTnI, we have modeled the relationship between hs-cTnT and hs-cTnI concentration using linear regression, allowing the user to employ the *T2-risk* score when either assay is available (Supplemental Figure 2).

Using the *T2-risk* score as a continuous measure, risk groups were defined in the derivation cohort for the primary and secondary outcomes, using lower and upper quartiles to delineate low and high risk, respectively, with the remainder forming an intermediate-risk group. These *T2-risk* thresholds were applied to the validation cohorts without recalibration. A further model was created in the derivation cohort excluding all outcome events in the first 30 days to account for critical illness and externally validated in both cohorts. All analyses were undertaken using deidentified data within a secure National Health Service Safe Haven (DataLoch) using R version 3.6.3 (R Foundation for Statistical Computing). All findings are reported in accordance with the Transparent Reporting of a Multivariable Prediction Model for Individual Prognosis or Diagnosis statement (Supplemental Appendix).

## Results

### Study populations

**Figure 1:**
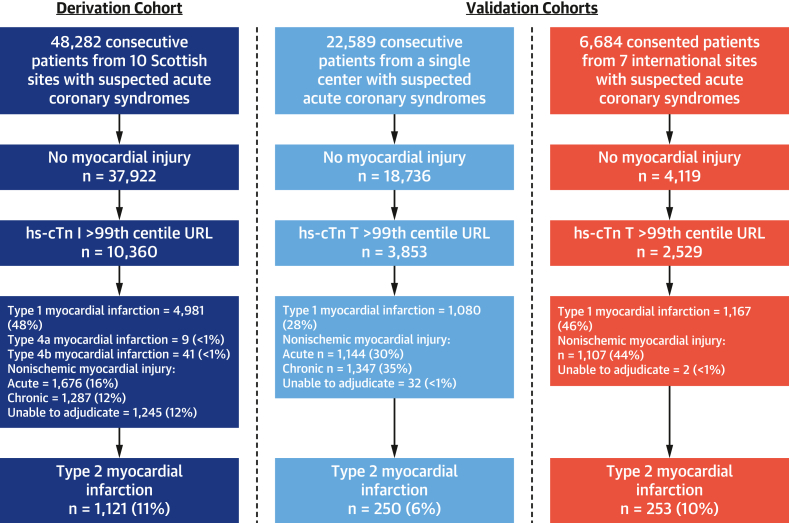
Flowchart of Study Populations Patient flowchart identifying participants in the derivation and single-center and multicenter validation cohorts. In the consented validation cohort, there were 36 patients in whom no troponin result was obtained. hs-cTn T = high-sensitivity cardiac troponin T; URL = upper reference limit.

The derivation cohort was identified from 48,282 consecutive patients with suspected acute coronary syndrome, of whom 21% (10,360) had myocardial injury. Sufficient clinical information was available to adjudicate the diagnosis in 88% (9,115 of 10,360) of patients. The final diagnosis was type 2 myocardial infarction in 12% (1,121 of 9,115), with 2 outliers excluded, giving a total of 1,119 patients for the derivation cohort. The single-center consecutive patient cohort was derived from 22,589 consecutive patients with suspected acute coronary syndrome, of whom 17% (3,853) had myocardial injury. The final diagnosis was type 2 myocardial infarction in 7% (250 of 3,853) of patients**.** The multicenter, international cohort was identified from 6,684 patients with suspected acute coronary syndrome. Of these, 38% (2,529) had evidence of myocardial injury, with type 2 myocardial infarction in 10% (253 of 2,529) (Figure 1, Supplemental Table 2).

Patients with type 2 myocardial infarction were of similar age in all cohorts (derivation cohort: 74 ± 13 years; single-center validation cohort: 72 ± 13 years; and multicenter validation cohort: 69 ± 14 years). A similar proportion of men and women were recruited in the consecutive patient cohorts (derivation 45% men, single-center validation 50% men); however, a higher proportion of men were enrolled in the multicenter, international validation cohort (65%). Patients in the derivation and multicenter, international validation cohorts had a higher incidence of cardiovascular disorders such as ischemic heart disease, previous myocardial infarction, and cerebrovascular disease than the single-center validation cohort. Physiological parameters were broadly comparable (Table 1).

**Table 1:** Baseline Characteristics of the Derivation and Validation Cohorts

	Derivation Cohort (n = 1,121)	Single-Center Validation Cohort (n = 250)	Multicenter Validation Cohort (n = 253)
Demographics			
Age, y	74 ± 14	72 ± 13	69 ± 14
Male	501 (45)	124 (50)	164 (65)
Medical history			
Myocardial infarction	163 (15)	48 (19)	69 (27)
Ischemic heart disease	454 (40)^a^	68 (27)^b^	105 (42)^a^
Cerebrovascular disease	135 (12)	20 (8)	22 (9)
Diabetes mellitus	147 (13)	56 (22)	67 (26)
Heart failure hospitalization	292 (26)	40 (16)	21 (8)
Anemia	398 (36)	100 (40)	63 (25)
Electrocardiogram			
Myocardial ischemia	383 (37)	68 (27)	104 (41)
Physiological parameters			
Heart rate, beats/min	105 ± 35	95 ± 31	100 ± 39
Systolic blood pressure, mm Hg	132 ± 30	141 ± 35	138 ± 31
Hematology and clinical chemistry			
Hemoglobin, g/L	126 ± 29	126 ± 27	137 ± 22
eGFR, mL/min/1.73 m^2^	64 ± 25	65 ± 25	70 ± 28
Peak hs-cTnI, ng/L	125 (48-604)	–	–
Peak hs-cTnT, ng/L	–	78 (34-177)	43 (26-87)
Mechanism of myocardial injury			
Coronary mechanisms	35 (3)	1 (<1)	20 (8)
Hypoxia	219 (20)	42 (17)	13 (5)
Anemia	95 (9)	28 (11)	14 (6)
Severe hypertension	61 (5)	22 (9)	47 (19)
Tachyarrhythmia	616 (55)	113 (45)	137 (54)
Hypotension	89 (8)	22 (9)	9 (4)

Values are mean ± SD, n (%), or median (IQR). Estimated glomerular filtration rate (eGFR) was calculated according to the Modification of Diet in Renal Disease equation. For 3 patients in the multicenter validation cohort, the mechanism could not be determined.

cTnI = high-sensitivity cardiac troponin I; hs-cTnT = high-sensitivity cardiac troponin T.

a Defined as prior angina, myocardial infarction, or revascularization.

b Defined as prior myocardial infarction or revascularization.

### Primary and secondary outcomes

The primary outcome of myocardial infarction or all-cause death at 1 year occurred in 27% (297 of 1,119) of the derivation cohort (Supplemental Table 3) and in 26% (66 of 250) and 14% (35 of 253) of the single-center and multicenter validation cohorts, respectively. The secondary outcome of myocardial infarction or cardiovascular death occurred in 14% (162 of 1,119) in the derivation cohort and 10% (26 of 250) and 10% (15 of 253) in the single-center and multicenter validation cohorts (Table 2).

**Table 2:** Outcome Events in Each Cohort at 1 Year

	Derivation Cohort (n = 1,121)	Single Center Validation Cohort (n = 250)	Multicenter Validation Cohort (n = 253)
Primary outcome			
Myocardial infarction or all-cause death	297 (27)	66 (26)	35 (14)
Secondary outcomes			
Myocardial infarction	49 (4)	13 (5)	12 (5)
All-cause death	258 (23)	60 (24)	28 (11)
Cardiovascular death	120 (11)	19 (8)	15 (6)
Noncardiovascular death	138 (12)	41 (16)	13 (5)

Values are n (%).

### Derivation of the *T2-risk* score

**Figure 2:**
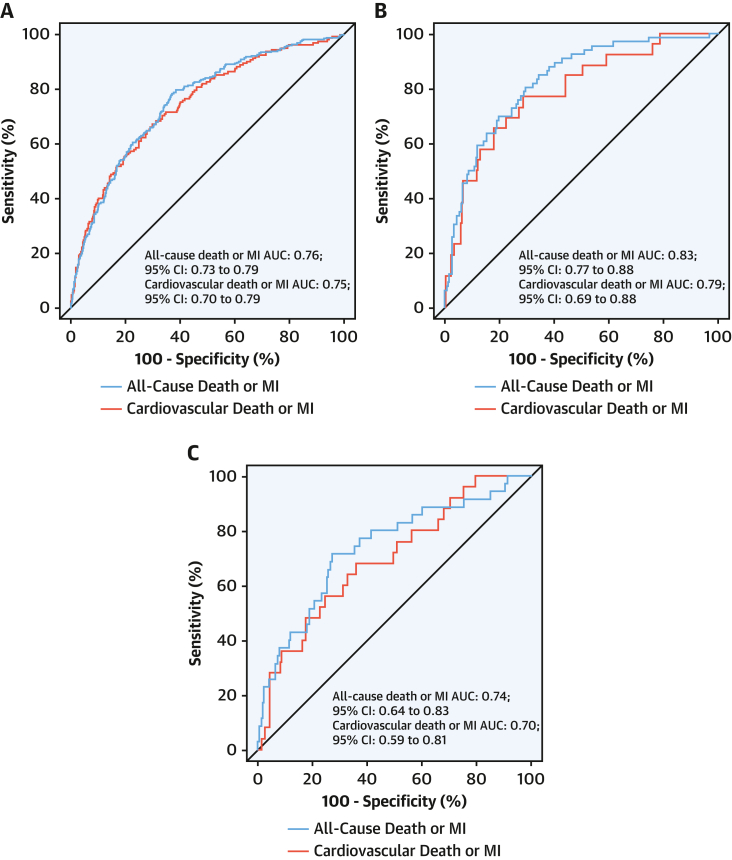
AUC for Outcomes for *T2-risk Score* Discrimination of the *T2-risk* model was assessed by using area under the receiving-operating characteristic curve (AUC) for the *T2-risk* model for the primary outcome of all-cause death or myocardial infarction (MI) at 1 year **(blue line)** and secondary outcome of cardiovascular death or MI at 1 year **(red line)** in the derivation cohort **(A)** and in the single-center, consecutive patient **(B)** and multicenter **(C)** validation cohorts.

**Figure 3:**
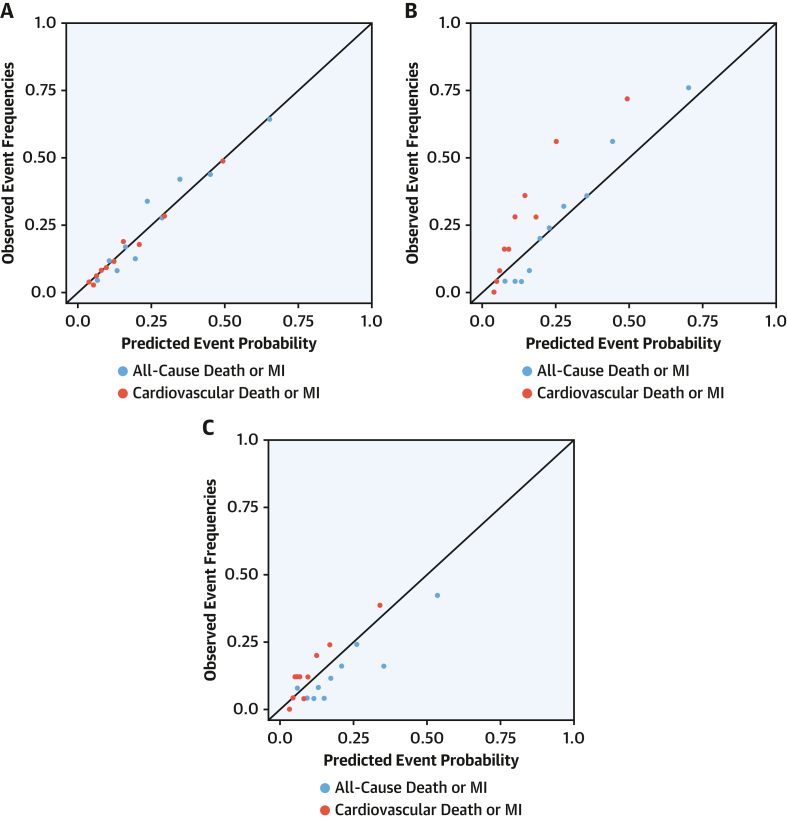
Calibration Plots for Outcomes of the *T2-risk* Score Calibration plot of the observed and predicted event rates in the derivation cohort **(A)** and in the single-center, consecutive patient **(B)** and multicenter **(C)** validation cohorts. Each **dot** represents one decile of risk. **Blue dots** are for primary outcome and **red dots** for the secondary outcome. Abbreviations as in Figure 2.

Univariate and multivariate HRs were derived for the primary outcome of myocardial infarction or all-cause death at 1 year (Table 3) and secondary outcome of myocardial infarction or cardiovascular death at 1 year (Supplemental Figure 3). For the primary outcome, the *T2-risk* score displayed good discrimination (AUC: 0.76; 95% CI: 0.73-0.79) (Figure 2A). For the secondary outcome, discrimination was similar (AUC: 0.75; 95% CI: 0.70-0.79). Calibration plots of the observed and predicted risk revealed that calibration was good (Figure 3A). Further assessment using bootstrapped bias-corrected calibration curves produced similar results (Supplemental Figure 4). We compared the *T2-risk* prediction tool vs the GRACE 2.0 algorithm for the primary outcome, which showed improved discrimination in the derivation cohort (AUC of 0.76 [95% CI: 0.73-0.79] vs AUC of 0.71 [95% CI 0.67-0.74; *P* = 0.038]) and both validation cohorts (Supplemental Table 4).

**Table 3:** Univariate and Multivariate Analysis of Covariates in the *T2-risk* Score

	Univariable HR (95% CI)	Multivariate HR (95% CI)
Anemia	2.21 (1.76-2.78)	1.52 (1.19-1.95)
Heart failure hospitalization	2.20 (1.75-2.78)	1.53 (1.19-1.96)
Previous myocardial infarction	1.89 (1.45-2.48)	
Diabetes mellitus	1.87 (1.41-2.49)	1.36 (0.99-1.86)
Ischemic heart disease	1.78 (1.42-2.24)	1.09 (0.84-1.40)
Cerebrovascular disease	1.76 (1.31-2.37)	
Age, y^a^	1.68 (1.21-2.33)	1.67 (1.19-2.34)
Myocardial ischemia on ECG	1.40 (1.11-1.75)	1.18 (0.93-1.50)
Log peak hs-cTnI, ng/L^b^	1.35 (1.16-1.57)	1.32 (1.12-1.55)
T-wave inversion on ECG	1.31 (0.98-1.75)	
Primary presentation with chest pain	1.30 (1.01-1.65)	
ST-segment deviation on ECG	1.21 (0.94-1.54)	
Male	0.96 (0.76-1.21)	
LBBB	0.96 (0.77-1.21)	
Heart rate, beats/min^c^	0.86 (0.71-1.04)	0.90 (0.74-1.10)
Atrial fibrillation	0.84 (0.66-1.07)	
eGFR, mL/min/1.73 m^2^^d^	0.63 (0.54-0.73)	0.89 (0.75-1.05)

Continuous covariate effects are modeled based on the upper vs lower interquartile range.

ECG = electrocardiogram; LBBB = Left bundle branch block; other abbreviations as in Table 1.

a Age 84 vs 67 years.

b hs-cTnI 602 vs 48 ng/L.

c 126 vs 79 beats/min.

d eGFR 81 vs 45 mL/min/1.73 m^2^.

### External validation of the *T2-risk* score

In the single-center, consecutive patient validation cohort, discrimination was similar for both the primary (AUC: 0.83; 95% CI: 0.77-0.87) and secondary (AUC: 0.79; 95% CI: 0.69-0.88) outcomes. In the multicenter, international validation cohort, discrimination was similar for the primary outcome (AUC: 0.74; 95% CI: 0.64-0.83) and secondary outcome (AUC: 0.70; 95% CI: 0.59-0.81) (Figures 2B and 2C). Calibration was assessed externally in both cohorts, with the predicted risk resembling the observed risk for both primary and secondary outcomes (Figures 3B and 3C).

### Risk stratification in patients with type 2 myocardial infarction

**Figure 4:**
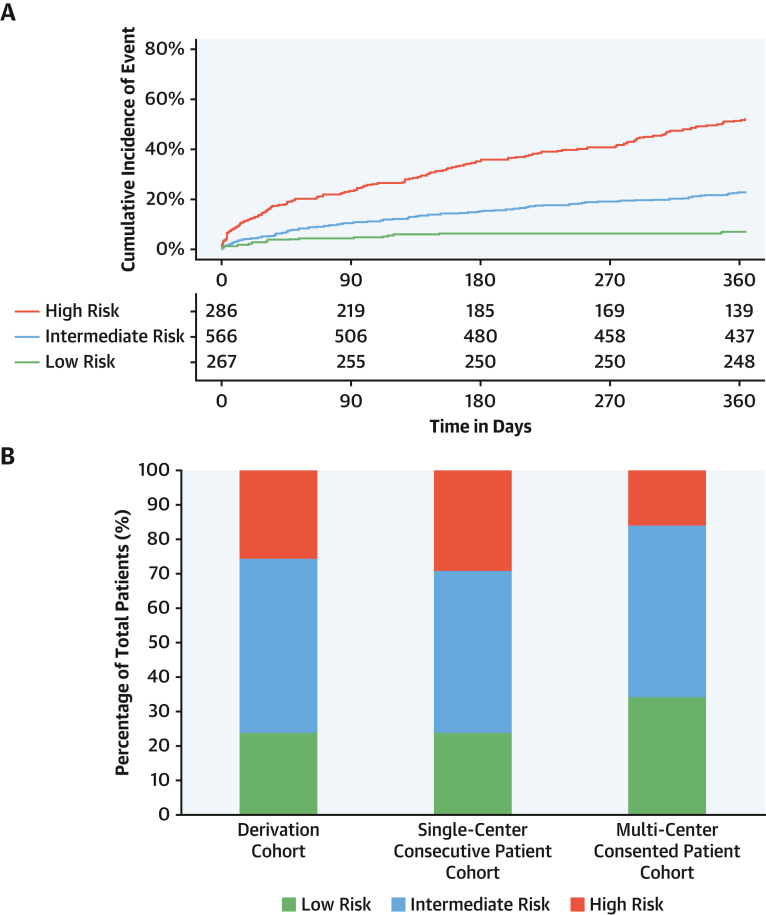
Cumulative Incidence of Events and Proportions of Risk Groups **(A)** Cumulative incidence plot of probability of primary outcome events at 1 year stratified according to risk group in the derivation cohort, adjusted for the competing risk of noncardiovascular death. **(B)** Stacked bar chart showing the proportion of patients stratified in risk groups according to the lower and upper quartiles in the derivation cohort.

When evaluated as a continuous variable, the upper quartile of the *T2-risk* score was applied to define patients at high risk (predicted event rate >34%) and the lower quartile applied to define patients at low risk (predicted event rate <13%). In the derivation cohort, the observed rate of subsequent myocardial infarction or death was 51% (147 of 286) at 1 year in those identified as high risk and 7% (19 of 267) in those at low risk (Figure 4). The characteristics of these patients are presented in Supplemental Table 5. These thresholds were applied to both validation cohorts without recalibration. In the single-center, consecutive patient cohort, 26% (65 of 250) and 24% (59 of 250) of patients were identified as high or low risk, with an observed event rate of 60% (39 of 65) and 3% (2 of 59) at 1 year, respectively. In the multicenter consented patient cohort, 16% (40 of 253) and 34% (86 of 253) of patients were identified as high or low risk, with an observed event rate of 35% (14 of 40) and 5% (4 of 86) at 1 year (Figure 4, Supplemental Figure 5). Risk thresholds for the secondary outcome performed similarly in the derivation cohort, with an observed event rate of 32% (88 of 272) in the high-risk category and 4% (9 of 252) in the low-risk category (Supplemental Figure 6).

An app has been developed to facilitate evaluation of the primary outcome model with prespecified risk thresholds.^27^

### Sensitivity and subgroup analyses

When primary outcome events up to 30 days were excluded to account for critical illness, the model performance was similar in both the derivation (AUC: 0.77; 95% CI: 0.73-0.80) and validation (AUC: 0.80 [95% CI: 0.72-0.87], single-center; AUC: 0.73 [95% CI: 0.62-0.85], multicenter]) cohorts, respectively. To ensure imputation did not influence results, a complete case analysis was undertaken, showing no difference in model performance (Supplemental Table 6). The model performed well when stratified according to sex (AUC: 0.73 [95% CI: 0.69-0.78] in women; AUC: 0.78 [95% CI: 0.74-0.83] in men), but the inclusion of sex did not improve discrimination. Performance of the *T2-risk* score was also evaluated in subgroups stratified according to etiology of type 2 myocardial infarction: coronary (AUC: 0.62; 95% CI: 0.26-0.98 [n = 35]), systemic (AUC: 0.72; 95% CI: 0.67-0.77 [n = 464]), and tachyarrhythmia (AUC: 0.76; 95% CI: 0.71-0.80 [n = 616]) phenotype. A systematic assessment of available covariates for the prediction of the primary outcome is shown in Table 3.

## Discussion

We have derived and externally validated a risk stratification tool to guide prognostication in patients with type 2 myocardial infarction. We show that routinely recorded clinical variables can be used to estimate the likelihood of subsequent myocardial infarction and death from any cause or death from cardiovascular disease at 1 year (Central Illustration). Compared with the established GRACE 2.0 algorithm, the *T2-risk* model showed improved performance for the prediction of myocardial infarction or death from any cause. Importantly, the *T2-risk* score performed well in 2 independent external validation cohorts and displayed consistent performance in men and women. In the future, this tool may be helpful for clinicians in practice to identify patients at highest risk of future cardiovascular events.

**Central Illustration:**
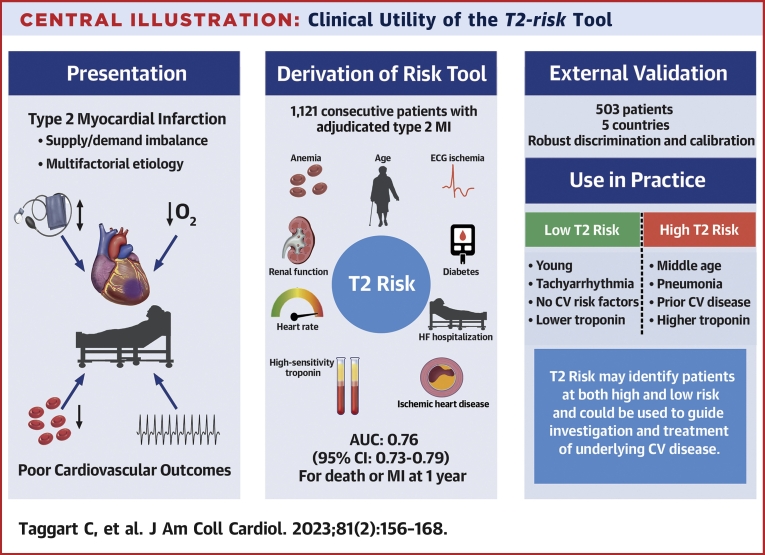
Clinical Utility of the *T2-risk* Tool How the *T2-risk* tool can be integrated into clinical care. **(Left)** Identification of type 2 myocardial infarction. **(Middle)** Derivation of the *T2-risk* model, its discriminatory performance, and clinical covariates. **(Right)** Validation of the *T2-risk* model and a case-based example.

Prior research has shown that the guideline-recommended GRACE 2.0 algorithm performs less well in patients with type 2 myocardial infarction than in those with type 1 myocardial infarction.^28^ Given that GRACE was derived in a population of patients with acute coronary syndrome identified using contemporary troponin assays who were recruited between 1999 and 2009, it is very likely that patients with type 2 myocardial infarction were not represented. Although attempts have been made to derive risk stratification tools in type 2 myocardial infarction, to date, these have been limited in scope and not widely applied. The TARRACO (Troponin Assessment for Risk Stratification of Patients Without Acute Coronary Atherothrombosis) risk score was derived and validated in patients with type 2 myocardial infarction and myocardial injury; it showed good discrimination for the prediction of death, myocardial infarction, or readmission for heart failure with an AUC of 0.71 (95% CI: 0.65-0.78).^29^ TARRACO performed less well when independently validated for an outcome of death or myocardial infarction (AUC: 0.47; 95% CI: 0.41-0.53).^30^ However, this validation did not include heart failure as an outcome, and events were assessed at 30 and 90 days rather than at 180 days as in the original derivation, which may in part explain differences in performance. These observations emphasize the importance of external validation, particularly in a condition as heterogeneous as type 2 myocardial infarction.

The *T2-risk* score facilitates identification of patients at highest risk of future myocardial infarction or death. Although we trained the *T2-risk* score for this outcome to enable a direct comparison with the GRACE 2.0 score, heart failure is also an important outcome for patients with type 2 myocardial infarction, and it is likely that risk prediction could be further refined with cardiac imaging. In patients with type 2 myocardial infarction, coronary disease and left ventricular dysfunction are highly prevalent. In a prospective cohort study enrolling patients with type 2 myocardial infarction, coronary artery disease was identified in 68% of patients and was obstructive in 30%.^31^ Importantly, this was previously unrecognized in more than one-half, with fewer than one-third of patients taking an antiplatelet and a statin. Similarly, evidence of left ventricular systolic dysfunction was identified in 34% of patients, and fewer than one-third were taking an angiotensin-converting enzyme inhibitor or a beta-blocker. The majority of events in the derivation cohort were attributable to cardiovascular causes, which may occur due to underlying untreated cardiac disease. Whether the *T2-risk* score could be used to target further investigation such as echocardiography, computed tomography imaging, or invasive coronary angiography to guide secondary prevention therapy requires prospective evaluation.

Although patients with type 2 myocardial infarction are at a higher overall risk of adverse outcomes, there are phenotypes of patients who are at lower risk such as those presenting with type 2 events secondary to tachyarrhythmia.^32^ Another application of the *T2-risk* score could be in the identification of those at low risk of future adverse outcomes who may not require further investigation. In the consecutive patient validation cohort, our risk thresholds identified similar proportions of patients at high and low risk of future events. We acknowledge that the proportion of high- and low-risk patients differed in the multicenter, international cohort. This likely reflects the recruitment of a lower risk population, as individual patient consent was sought to obtain additional research samples for storage. However, despite variation in the prevalence of type 2 myocardial infarction and risk profile of those enrolled across our validation cohorts, the *T2-risk* model performed consistently well.

### Study strengths

Our analysis has several strengths. The *T2-risk* score was developed from a large consecutive patient cohort, including all who presented to the hospital with suspected acute coronary syndrome, avoiding selection bias and including the largest number of consecutive patients with an adjudicated diagnosis of type 2 myocardial infarction. The score was validated in 2 independent patient cohorts encompassing 11 sites in 5 countries across Europe in both consecutive and selected patients. In all cohorts, the diagnosis of myocardial infarction was adjudicated in line with the fourth universal definition using high-sensitivity cardiac troponin assays.^1^ We applied routinely available clinical covariates to maximize simplicity of use for clinical practice and have made our model available without license to encourage further validation in other health care settings.

### Study limitations

Although the GRACE 2.0 algorithm was derived in a global registry, the *T2-risk* score was derived in patients who were resident in Scotland and treated in a single health care system. However, we provide external validation in 2 independent cohorts showing that our findings are generalizable. To mitigate for competing risks in the prediction of future cardiovascular events in the secondary model, we censored for noncardiovascular death. Although this approach has been validated, this may lead to overprediction of secondary outcomes.^33^ We did not evaluate the performance of the *T2-risk* model in patients with acute nonischemic myocardial injury, but these patients share similar characteristics and outcomes, and it would be of interest to evaluate in future studies. The derivation and validation cohorts used the hs-cTnI and hs-cTnT assay, respectively, but we modeled the relationship in patients with both concentrations measured and applied a linear regression correction, revealing robust performance. We used multiple imputation for missing variables in the derivation cohort that were presumed to be at random, and showed in a sensitivity analysis that similar performance was observed in the complete data set without imputation. We were not able to incorporate all physiological parameters nor include phenotypic information known to influence prognosis. Although all index and outcome events were adjudicated in the derivation cohort and the multicenter validation cohort, ICD-10 coding was used for outcomes in the single-center validation cohort, which could contribute to misclassification and underestimate *T2-risk* model performance. Finally, although our risk stratification tool seems to delineate risk well, prospective evaluation is required if it is to be used to guide treatment decisions in practice.

## Conclusions

A novel risk stratification tool predicts myocardial infarction and death from any cause or cardiovascular disease at 1 year with good discrimination in patients with type 2 myocardial infarction. The *T2-risk* score performs well in different health care settings and could help clinicians prognosticate, as well as target investigation and preventative therapies more effectively.

Perspectives **COMPETENCY IN PATIENT CARE AND PROCEDURAL SKILLS:** A stratification tool derived and externally validated in patients with type 2 myocardial infarction improves prediction of re-infarction or death at 1 year.

**TRANSLATIONAL OUTLOOK:** Whether management guided by this risk stratification tool can improve outcomes in patients with type 2 myocardial infarction at highest risk of recurrent ischemic events requires prospective investigation.

## Funding Support and Author Disclosures

This project was funded by a Starter Grant for Clinical Lecturers from the Academy of Medical Sciences (ARC; SGL021/1075). This work was also supported by DataLoch, which is funded by the Data Driven Innovation programme within the Edinburgh and Southeast Scotland City Region Deal. The High-STEACS trial was funded by a Special Project Grant (SP/12/10/29922) from the British Heart Foundation with assay reagent, calibrators, and controls provided by Abbott Laboratories without charge. Dr Taggart is supported by a British Heart Foundation Clinical Research Training Fellowship (FS/CRTF/21/2473). Dr Monterrubio-Gómez is supported by an MRC University Unit grant to the MRC Human Genetics Unit. Dr Vallejos is a Chancellor’s Fellow supported by the University of Edinburgh. Drs Wereski and Bularga are supported by Clinical Research Training Fellowships from the Medical Research Council (MR/V007017/1 and MR/V007254/1). Dr Mills is supported by a Chair Award, Programme Grant, Research Excellence Award (CH/F/21/90010, RG/20/10/34966, RE/18/5/34216), from the British Heart Foundation, Drs Vallejos and Mills are supported by a British Heart Foundation–Turing Cardiovascular Data Science Award (BCDSA/100003). Dr Mills has received honoraria or consultancy from Abbott Diagnostics, Roche Diagnostics, Siemens Healthineers, and LumiraDx. Dr Roos was supported by the Stockholm County Council (grant no. 20200935). Dr Boeddinghaus has received research grants from the University of Basel, the University Hospital of Basel and the Division of Internal Medicine, the Swiss Academy of Medical Sciences, the Gottfried and Julia Bangerter-Rhyner-Foundation, and the Swiss National Science Foundation; and has received speaker honoraria and/or consulting honoraria from Siemens, Roche, Ortho Clinical Diagnostics, and Quidel Corporation, outside of the submitted work.). Dr Nestelberger has received research support from the Swiss National Science Foundation (P400PM_191037/1), the Swiss Heart Foundation (FF20079), the Prof Dr Max Cloëtta Foundation, the Margarete und Walter Lichtenstein-Stiftung (3MS1038), the University of Basel, and the University Hospital Basel; and has received speaker honoraria/consulting honoraria from Siemens, Beckman Coulter, Bayer, Ortho Clinical Diagnostics, and Orion Pharma, all outside the submitted work. Dr Koechlin received a research grant from the Swiss Heart Foundation, the University of Basel, the Swiss Academy of Medical Sciences, and the Gottfried and Julia Bangerter-Rhyner Foundation, and the Freiwillige Akademische Gesellschaft Basel. All other authors have reported that they have no relationships relevant to the contents of this paper to disclose.

## References

[1] Thygesen K., Alpert J.S., Jaffe A.S. (2018). Fourth universal definition of myocardial infarction (2018). Eur Heart J.

[2] DeFilippis A.P., Chapman A.R., Mills N.L. (2019). Assessment and treatment of patients with type 2 myocardial infarction and acute nonischemic myocardial injury. Circulation.

[3] Shah A.S.V., Anand A., Strachan F.E. (2018). High-sensitivity troponin in the evaluation of patients with suspected acute coronary syndrome: a stepped-wedge, cluster-randomised controlled trial. Lancet.

[4] Chapman A.R., Shah A.S.V., Lee K.K. (2018). Long-term outcomes in patients with type 2 myocardial infarction and myocardial injury. Circulation.

[5] Lambrecht S., Sarkisian L., Saaby L., Poulsen T.S., Thygesen K., Mickley H. (2018). Different causes of death in patients with myocardial infarction type 1, type 2, and myocardial injury. Am J Med.

[6] Chapman A.R., Adamson P.D., Shah A.S.V. (2020). High-sensitivity cardiac troponin and the universal definition of myocardial infarction. Circulation.

[7] Singh A., Gupta A., DeFilippis E.M. (2020). Cardiovascular mortality after type 1 and type 2 myocardial infarction in young adults. J Am Coll Cardiol.

[8] Raphael C.E., Roger V.L., Sandoval Y. (2020). Incidence, trends, and outcomes of type 2 myocardial infarction in a community cohort. Circulation.

[9] Fox K.A.A., Dabbous O.H., Goldberg R.J. (2006). Prediction of risk of death and myocardial infarction in the six months after presentation with acute coronary syndrome: prospective multinational observational study (GRACE). Br Med J.

[10] Fox K.A.A., FitzGerald G., Puymirat E. (2014). Should patients with acute coronary disease be stratified for management according to their risk? Derivation, external validation and outcomes using the updated GRACE risk score. BMJ Open.

[11] Antman E.M., Cohen M., Bernink P.J.L.M. (2000). The TIMI risk score for unstable angina/non-ST elevation MI. JAMA.

[12] Collet J.-P., Thiele H., Barbato E. (2021). 2020 ESC guidelines for the management of acute coronary syndromes in patients presenting without persistent ST-segment elevation. Eur Heart J.

[13] Amsterdam E.A., Wenger N.K., Brindis R.G. (2014). 2014 AHA/ACC guideline for the management of patients with non-ST-elevation acute coronary syndromes: a report of the American College of Cardiology/American Heart Association Task Force on Practice Guidelines. J Am Coll Cardiol.

[14] National Institute for Clinical Excellence (NICE). Acute coronary syndromes. NICE guideline NG185 (2020), recommendations 1.2.7 and 1.2.10. 2020. Accessed November 28, 2022. https://www.nice.org.uk/guidance/NG185

[15] Lee K.K., Ferry A.V., Lee K.K. (2019). Sex-specific thresholds of high-sensitivity troponin in patients with suspected acute coronary syndrome. J Am Coll Cardiol.

[16] Kadesjö E., Roos A., Siddiqui A., Desta L., Lundbäck M., Holzmann M.J. (2019). Acute versus chronic myocardial injury and long-term outcomes. Heart.

[17] Roos A., Sartipy U., Ljung R. (2018). Relation of chronic myocardial injury and non-ST-segment elevation myocardial infarction to mortality. Am J Cardiol.

[18] Shah A.S.V., Griffiths M., Lee K.K. (2015). High sensitivity cardiac troponin and the under-diagnosis of myocardial infarction in women: prospective cohort study. BMJ.

[19] Giannitsis E., Kurz K., Hallermayer K., Jarausch J., Jaffe A.S., Katus H.A. (2010). Analytical validation of a high-sensitivity cardiac troponin T assay. Clin Chem.

[20] Reichlin T., Irfan A., Twerenbold R. (2011). Utility of absolute and relative changes in cardiac troponin concentrations in the early diagnosis of acute myocardial infarction. Circulation.

[21] Twerenbold R., Costabel J.P., Nestelberger T. (2019). Outcome of applying the ESC 0/1-hour algorithm in patients with suspected myocardial infarction. J Am Coll Cardiol.

[22] Wereski R., Kimenai D.M., Bularga A. (2022). Risk factors for type 1 and type 2 myocardial infarction. Eur Heart J.

[23] (2011). Haemoglobin concentrations for the diagnosis of anaemia and assessment of severity. Vitamin and Mineral Nutrition Information System. World Health Organization.

[24] Therneau B.Y.T.M., Grambsch P.M., Fleming T.R. (1990). Martingale-based residuals for survival models. Biometrika.

[25] Belsley D., Kuh E., Welsh R.E. (2004).

[26] Kooperberg C., Stone C.J., Truong Y.K. (1995). Hazard regression. J Am Stat Assoc.

[27] The University of Edinburgh T2 risk stratification tool for patients with type 2 myocardial infarction. https://t2score.shinyapps.io/t2_riskscore/.

[28] Hung J., Roos A., Kadesjö E. (2021). Performance of the GRACE 2.0 score in patients with type 1 and type 2 myocardial infarction. Eur Heart J.

[29] Cediel G., Sandoval Y., Sexter A. (2019). Risk estimation in type 2 myocardial infarction and myocardial injury: the TARRACO Risk Score. Am J Med.

[30] Murphy S.P., McCarthy C.P., Cohen J.A. (2020). Application of the GRACE, TIMI, and TARRACO risk scores in type 2 myocardial infarction. J Am Coll Cardiol.

[31] Bularga A., Hung J., Daghem M. (2022). Coronary artery and cardiac disease in patients with type 2 myocardial infarction: a prospective cohort study. Circulation.

[32] Bularga A., Taggart C., Mendusic F. (2022). Assessment of supply-demand imbalance and outcomes in patients with type 2 myocardial infarction. JAMA Open.

[33] Putter H., Fiocco M., Geskus R.B. (2007). Tutorial in biostatistics: competing risks and multi-state models. Stat Med.

