# Supplementary material for: Improving Risk Stratification for Patients With Type 2 Myocardial Infarction
Source: J Am Coll Cardiol. 2023 Jan 17;81(2):156–68. doi: 10.1016/j.jacc.2022.10.025 (PMC9841577; doi:10.1016/j.jacc.2022.10.025)
Supplement: Supplemental Data [file mmc1.docx]

SUPPLEMENTARY DATA

# Improving Risk Stratification for Patients With Type 2 Myocardial Infarction

Caelan Taggart MD a*, Karla Monterrubio-Gómez PhD b*, Andreas Roos MD PhD c,d,

Jasper Boeddinghaus MD a, Dorien M. Kimenai PhD a, Erik Kadesjo MD PhD c,e,

Anda Bularga MD a, Ryan Wereski MD a, Amy Ferry PhD a, Matthew Lowry MD a,

Atul Anand MD PhD a, Kuan Ken Lee MD a, Dimitrios Doudesis MSc a,f,

Ioanna Manolopoulou PhD g, Thomas Nestelberger MD h, Luca Koechlin MD h,

Pedro Lopez-Ayala MD h, Christian Mueller MD h, Nicholas L. Mills MD PhD a,f,

Catalina A. Vallejos PhD b,i, Andrew R. Chapman MD PhD a

*a. BHF Centre for Cardiovascular Science, University of Edinburgh, 49 Little France Crescent, Edinburgh, UK*

*b. MRC Human Genetics Unit, Institute of Genetics and Cancer, University of Edinburgh, Edinburgh, UK*

*c. Department of Medicine, Clinical Epidemiology Division, Karolinska Institute, Solna, Stockholm, Sweden.*

*d. Department of Emergency and Reparative Medicine, Karolinska University Hospital, Stockholm, Sweden*

*e. Department of Medicine, Karolinska Institute, Solna, Stockholm, Sweden*

*f. Usher Institute, University of Edinburgh, EH8 9AG Edinburgh, UK*

*g. Department of Statistical Sciences, University College London, UK*

*h. Cardiovascular Research Institute Basel (CRIB), University Hospital Basel, Switzerland*

*i. The Alan Turing Institute, London, UK*

** The first two authors contributed equally to the study*

**Correspondence and requests for reprints:**

Dr Andrew R Chapman

BHF Centre for Cardiovascular Science

Chancellors Building

Royal Infirmary of Edinburgh

Edinburgh EH16 4SA

United Kingdom

Tel: +44-131-242-6515

Fax: +44-131-242-6379

Email: a.r.chapman@ed.ac.uk

Twitter: @chapdoc1 @CaelanTaggart @HighSTEACS

**Study approvals**

The study was approved by the Scotland A Research Ethics Committee, the Public Benefit and Privacy Panel for Health and Social Care, and by each National Health Service Health Board. Individual patient consent was not required and data from consecutive patients was collected prospectively from the electronic record, deidentified and linked within secure National Health Service Safe Havens. In the Karolinska University Hospital cohort, the study protocol was approved by the Regional Ethical Review Board in Stockholm. Both studies were conducted in accordance with the Declaration of Helsinki. Computing for this project was performed on the Scottish National Safe Haven (NSH), supported by the electronic Data Research and Innovation Service (eDRIS), itself a subsidiary of Public Health Scotland, and the Edinburgh Parallel Computing Centre (EPCC), based at the University of Edinburgh.

**Patient and public involvement**

Patients and lay representatives were members of the steering committee for the trial and all related studies and were involved in the design, conduct and approval of the High-STEACS study.

**Adjudication processes**

All patients with hs-cTnI concentrations above the sex-specific 99th centile were classified according to the Third Universal Definition of Myocardial Infarction in use at the time of the trial. In this pre-specified secondary analysis, we updated this classification in accordance with the Fourth Universal Definition of Myocardial Infarction. The final diagnosis was adjudicated according to a pre-specified list (cardiac diagnoses: acute aortic dissection, acute heart failure, cardiomyopathy, chronic heart failure, hypertensive heart disease, myopericarditis, non-ST segment elevation myocardial infarction, ST-segment elevation myocardial infarction, recent myocardial infarction, tachyarrhythmia, Takotsubo cardiomyopathy or valvular heart disease; non-cardiac diagnoses: acute kidney injury, chronic kidney disease, chronic obstructive pulmonary disease, gastrointestinal bleed, pulmonary embolism, sepsis, or other). Two physicians independently reviewed all clinical information, blinded to study phase, with discordant diagnoses resolved by a third reviewer. Clinical information included the dates and times of presentation and final discharge, the initial emergency department assessment and final discharge letter as documented in the electronic care record, with summaries of all investigations undertaken during the index presentation including the electrocardiogram. The adjudication panel had access to raw clinical information including haemoglobin, creatinine, and high-sensitivity cardiac troponin I concentrations, and the reports from invasive coronary angiography. Type 1 myocardial infarction was defined as myocardial necrosis (any hs-cTnI concentration above the 99th centile with a rise and/or fall in hs-cTnI concentration where serial testing was performed) in the context of a presentation with suspected acute coronary syndrome with symptoms or signs of myocardial ischemia on the electrocardiogram. Patients with symptoms or signs of myocardial ischemia and evidence of increased oxygen demand or decreased supply (for example, tachyarrhythmia, hypotension, or anaemia) secondary to an alternative pathology and myocardial necrosis were defined as type 2 myocardial infarction. The classification of type 2 myocardial infarction also includes patients with coronary vasospasm, embolism or spontaneous dissection without evidence of atherothrombosis related to coronary artery disease. Type 4a myocardial infarction was defined in patients with symptoms or signs of myocardial ischemia following percutaneous coronary intervention where hs-cTnI concentrations were 5-fold greater than the 99th centile, or increased further if elevated prior to the procedure. Type 4b myocardial infarction was defined where myocardial ischemia and myocardial necrosis were associated with stent thrombosis documented at angiography. Myocardial injury was defined if hs-cTnI concentrations were above the 99th centile in the absence of any clinical features of myocardial ischemia. All non-ischemic myocardial injury was classified as acute, unless a change of <20% was observed on serial testing or the final adjudicated diagnosis was chronic heart failure or chronic renal failure, where the classification was chronic myocardial injury.

A similar process was used in the consecutive validation cohort (Sweden). Myocardial infarction (MI) was defined and cTn levels interpreted as recommended in current guidelines (1–3). In brief, MI was diagnosed when there was evidence of myocardial necrosis with a significant rise and/or fall in a clinical setting consistent with myocardial ischemia. Patients with MI were further subdivided into type 1 MI (primary coronary events) and type 2 MI (ischemia due to increased demand or decreased supply, for example tachyarrhythmia or hypertensive crisis).(1, 4) All other patients were classified in the categories of unstable angina (UA), Non-Cardiac Chest Pain (NCCP), cardiac but non-coronary disease (e.g., tachyarrhythmia, perimyocarditis), and symptoms of unknown origin with normal concentrations of hs-cTn.

In the multi-centre consented patient cohort (APACE), the adjudication of final diagnoses was performed centrally in the core lab (University Hospital Basel) for all patients incorporating levels of hs-cTnT. More specifically, two independent cardiologists not directly involved in patient care reviewed all available medical records (including patient history, physical examination, results of laboratory testing including hs-cTn levels, radiologic testing, ECG, echocardiography, cardiac exercise test, lesion severity and morphology in coronary angiography, discharge summary) pertaining to the patient from the time of ED presentation to 90-day follow-up. In situations of diagnostic disagreement, cases were reviewed and adjudicated in conjunction with a third cardiologist.

**
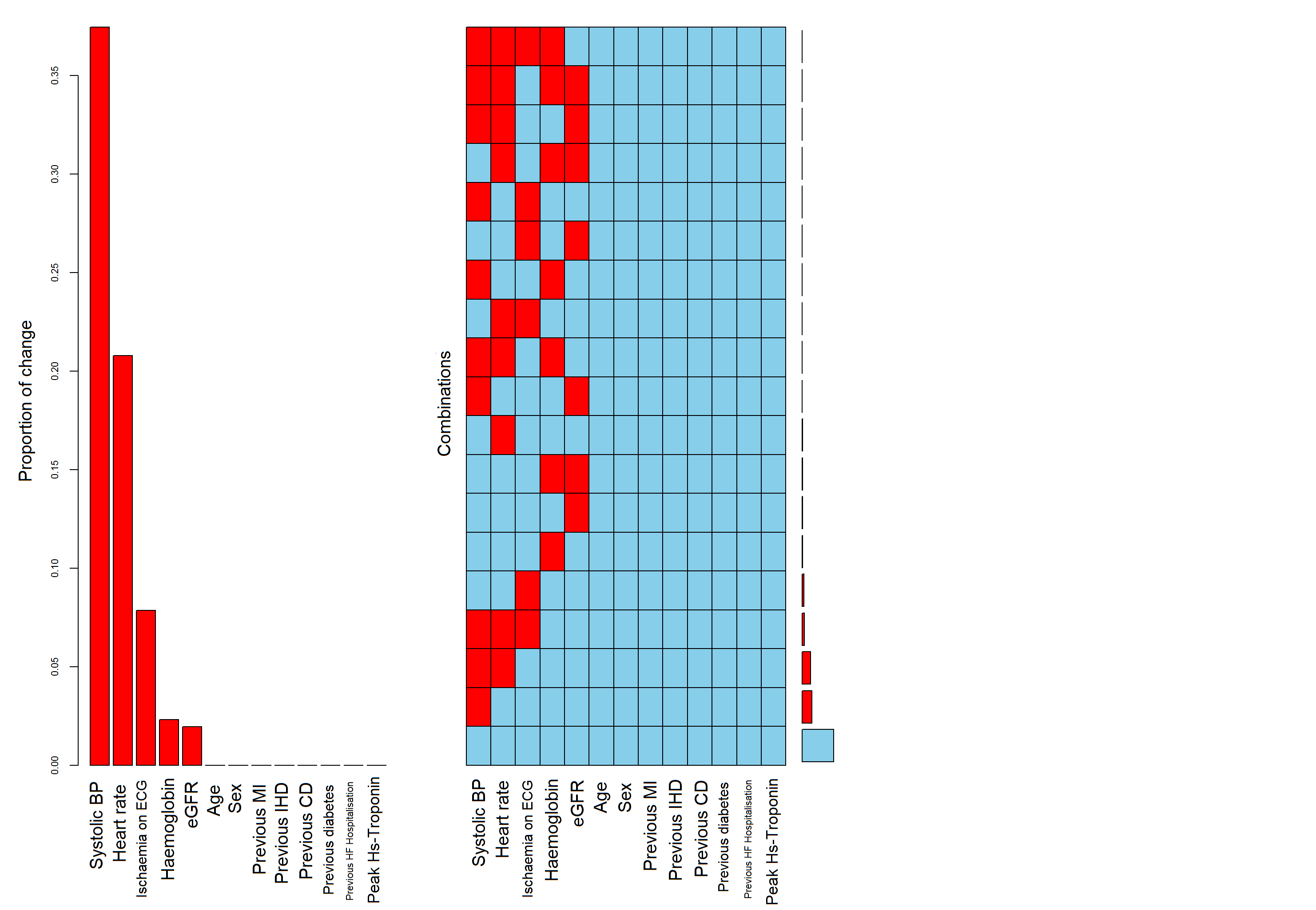

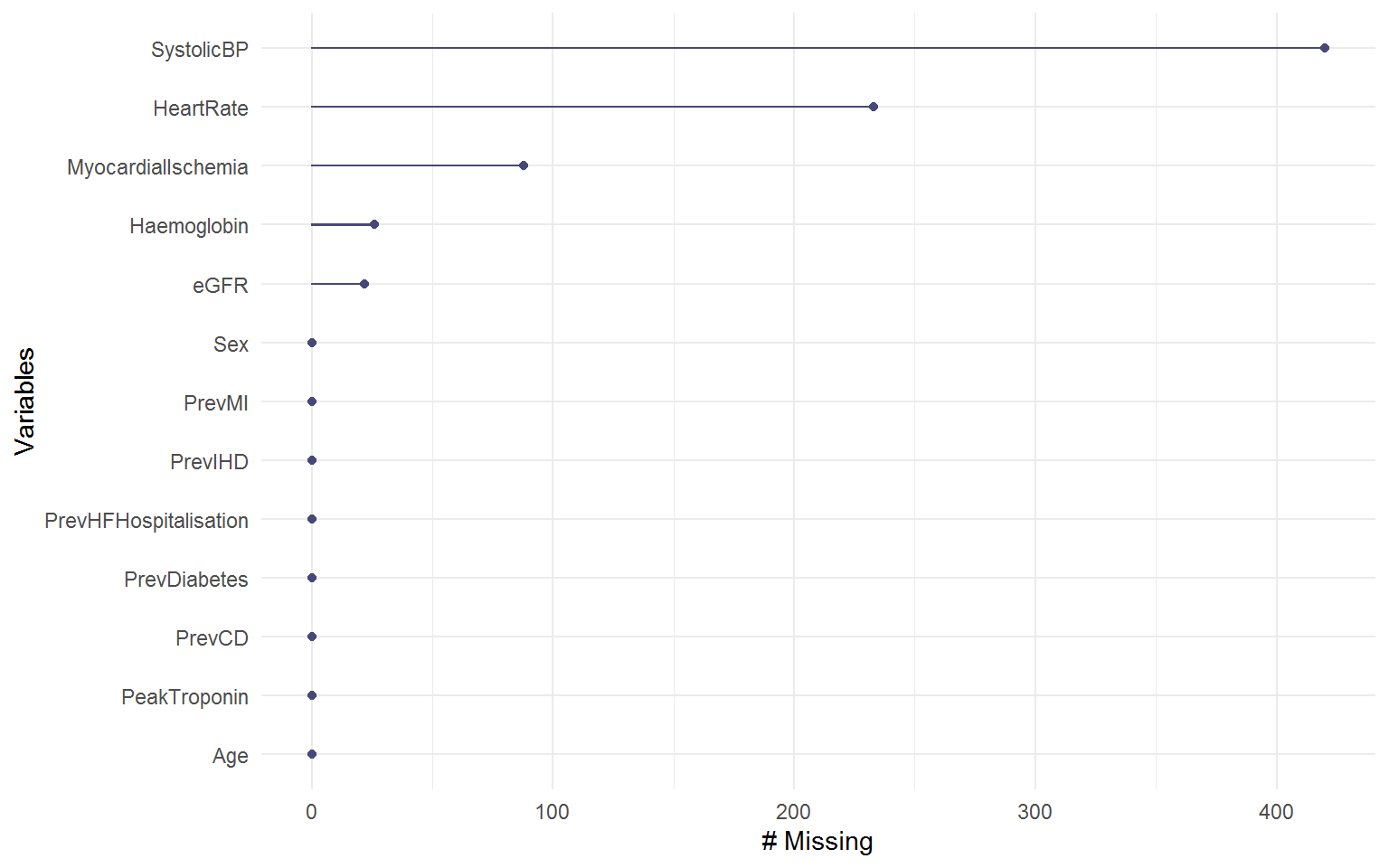
Figure 1: Missing values of the clinical covariates of interest in the T2-risk stratification tool and visualisation of randomness.**

(i)

(ii)

Figure (i) quantifies the number of missing values in the clinical co-variates of interest in the T2-risk tool. As the proportion of missing values for systolic blood pressure (Systolic BP) was very high (37.5%) this term was excluded. In order to impute missing values, the *mice* R package was employed, with predicting mean matching (pmm). This method works for both continuous and discrete covariates, ensuring that the imputed values have a similar distribution as those observed, and does not produce imputed values outside the range of the observed data. In addition, pmm is known for being robust to transformations (e.g., log). Figure (ii) illustrates no clear pattern of missingness of this data which allows us to safely assume that these are missing at random. PrevDiabetes = Previous Diabetes, MyocardialIschaemia = Myocardial ischaemia on electrocardiogram, eGFR = estimated glomerular filtration rate, PrevCD = previous cerebrovascular disease, PrevIHD = previous ischaemic heart disease, PreMI = previous myocardial infarction.

**Figure 2: Linear regression model for the relationship between of hs-cTnT and hs-cTnI.**

**
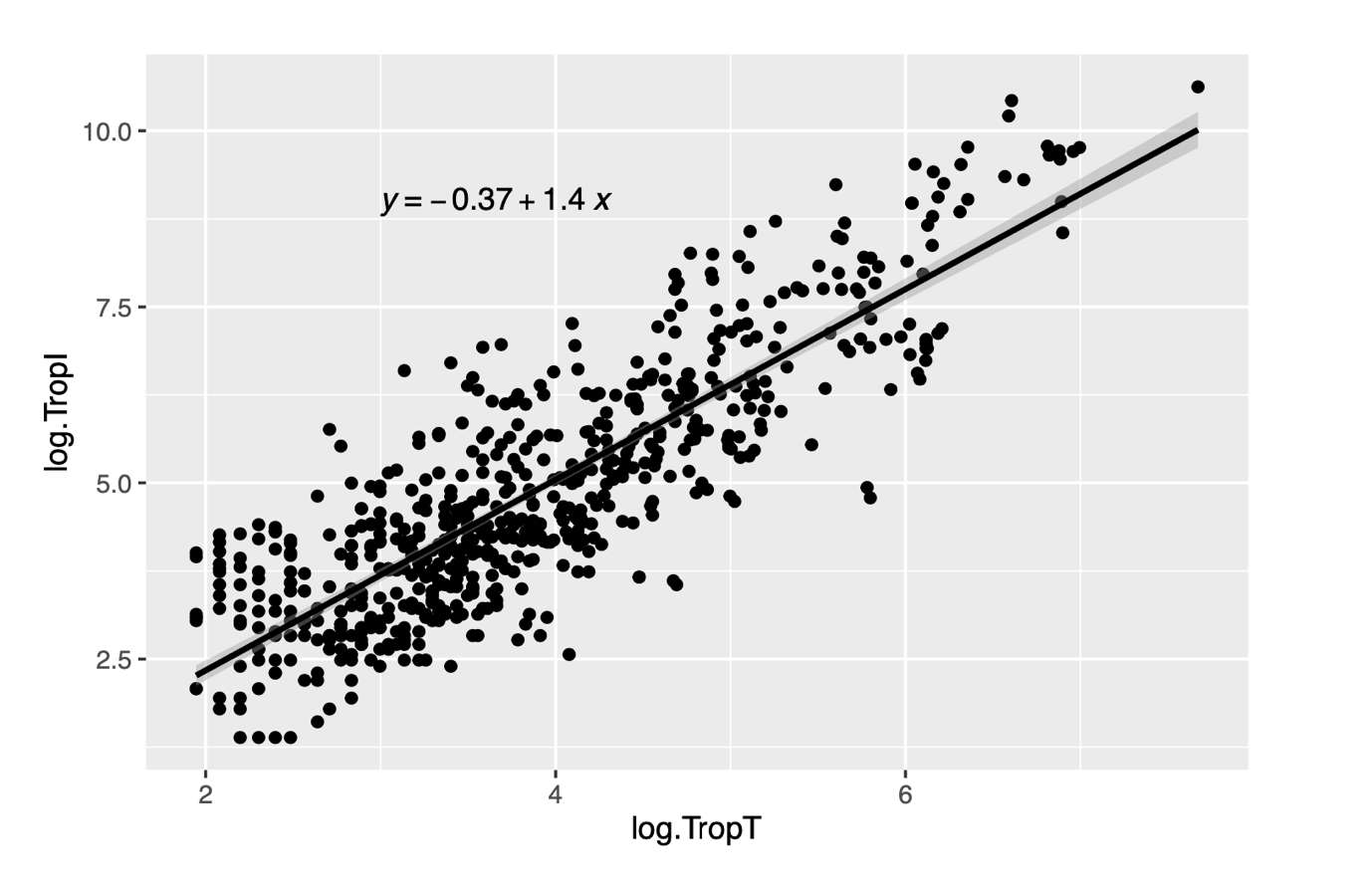
**

Estimates obtained from 610 patients within the High-STEACS trial with matched samples obtained. A linear function is included in the model to allow the inclusion of hs-cTnT for the calculation of the *T2-risk* score.

**
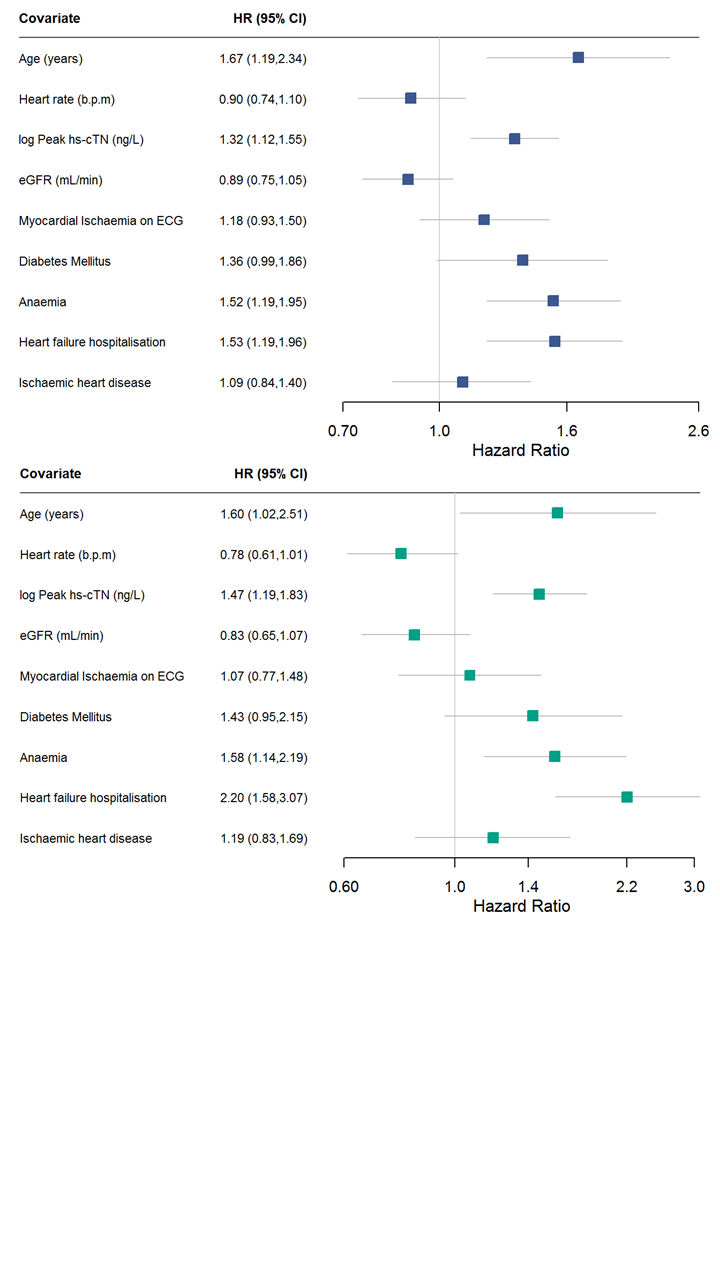
Figure 3: Forest plot of hazard ratios (95% CI) for each covariate on the primary outcome (blue) and secondary outcome (green).**

Continuous co-variate effects are modelled based on the upper versus lower interquartile range. Age, 84 *versus* 67 years, Heart rate, 126 *versus* 79 beats per minute, eGFR, 81 *versus* 45 ml/min, hs-cTnI 602 *versus* 48 ng/L.

**Figure 4: Observed versus predicted events for primary and secondary outcome models**


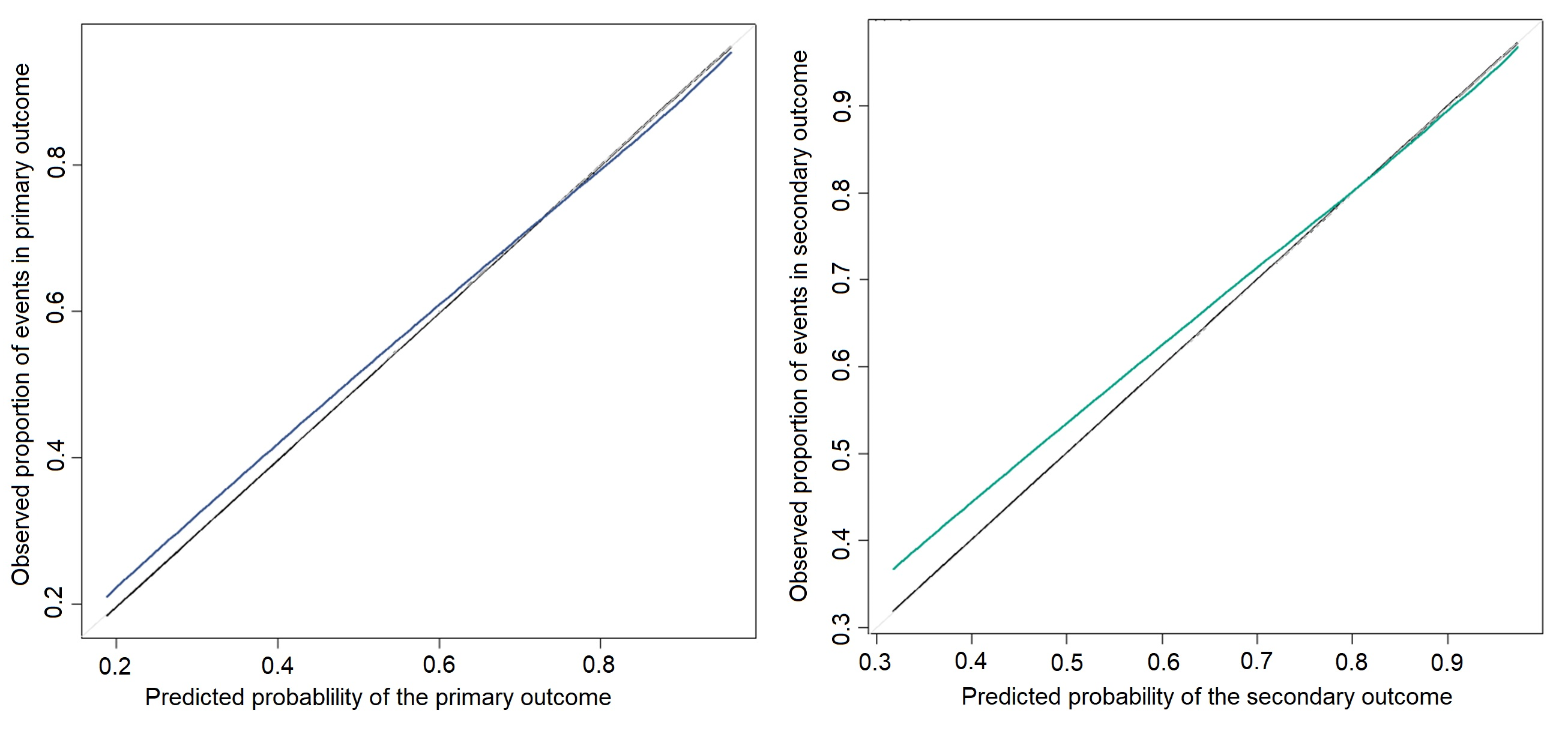


**Figure 5: Cumulative incidence of the primary outcome in low, intermediate and high-risk groups in the (i) single-centre consecutive and (ii) multi-centre international validation cohorts.**

**(ii)**

**(i)**

**
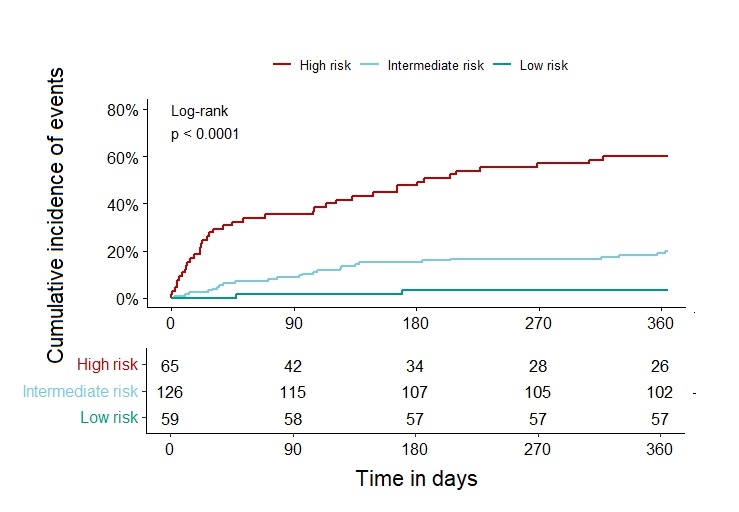

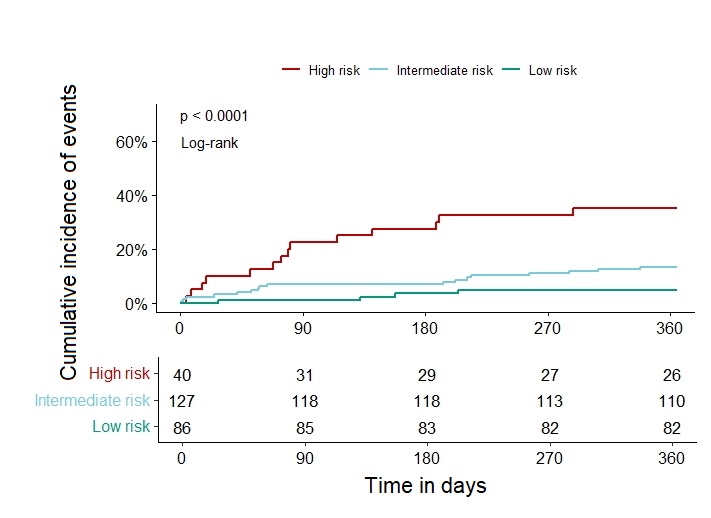
**

**Figure 6: Cumulative incidence of the secondary outcome of cardiovascular death or myocardial infarction at one year stratified by risk group in the derivation cohort**

**
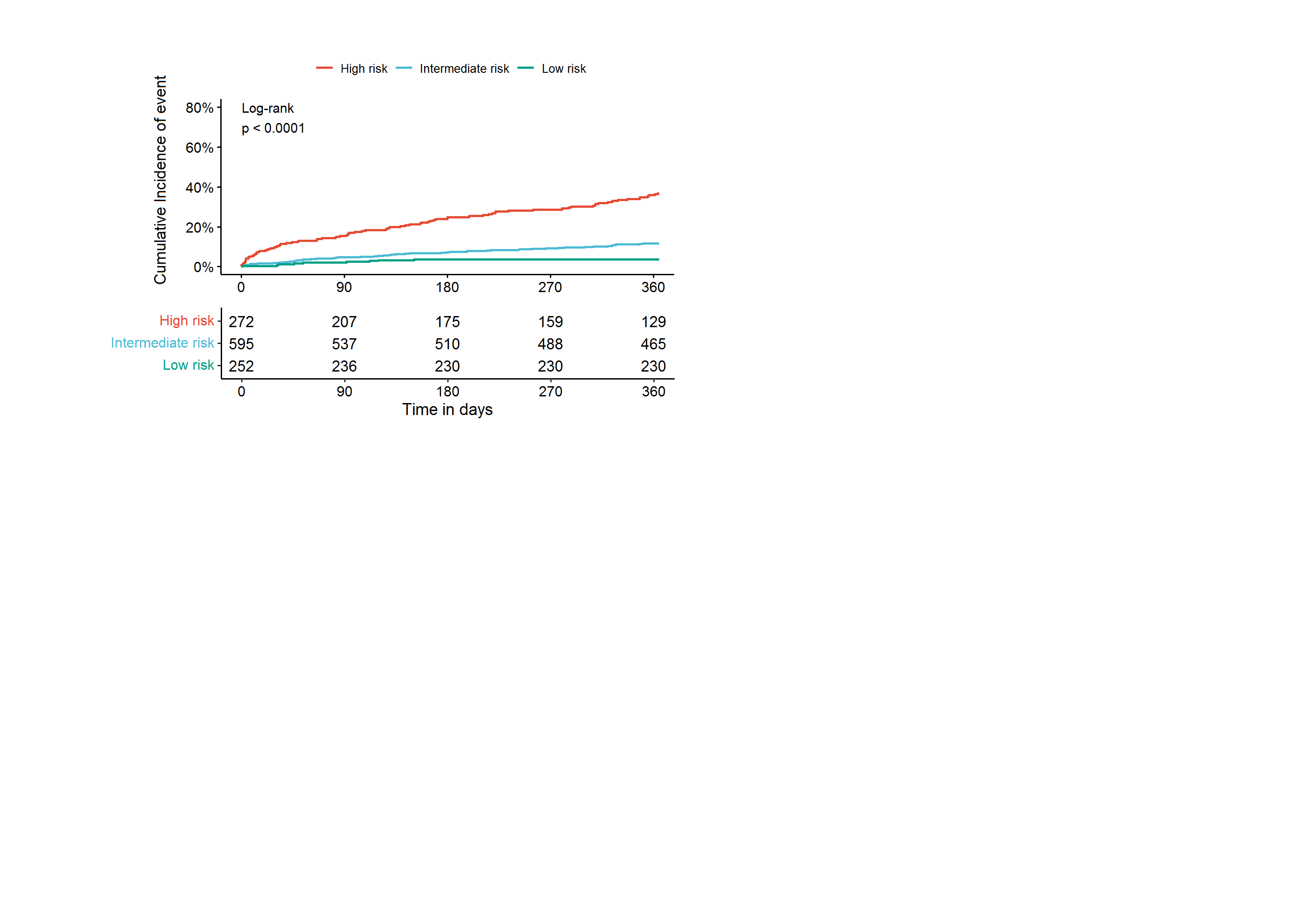
**

**Figure 7: Correlation matrix of Pearson correlation coefficients testing for co-linearity in co-variates available for the *T2-Risk* tool.**

**
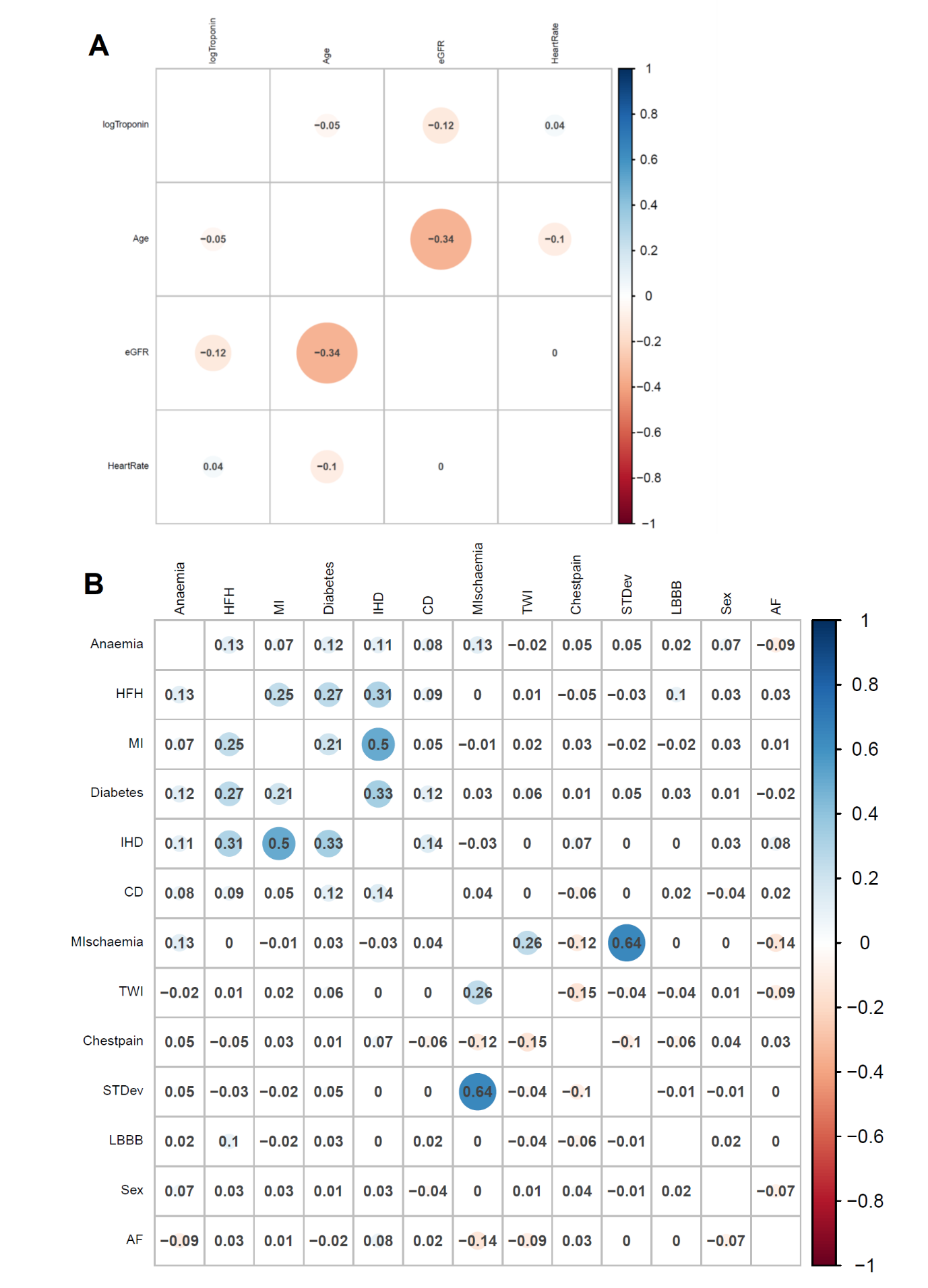
**

**Figure 8: Risk equation for the *T2-risk* tool**


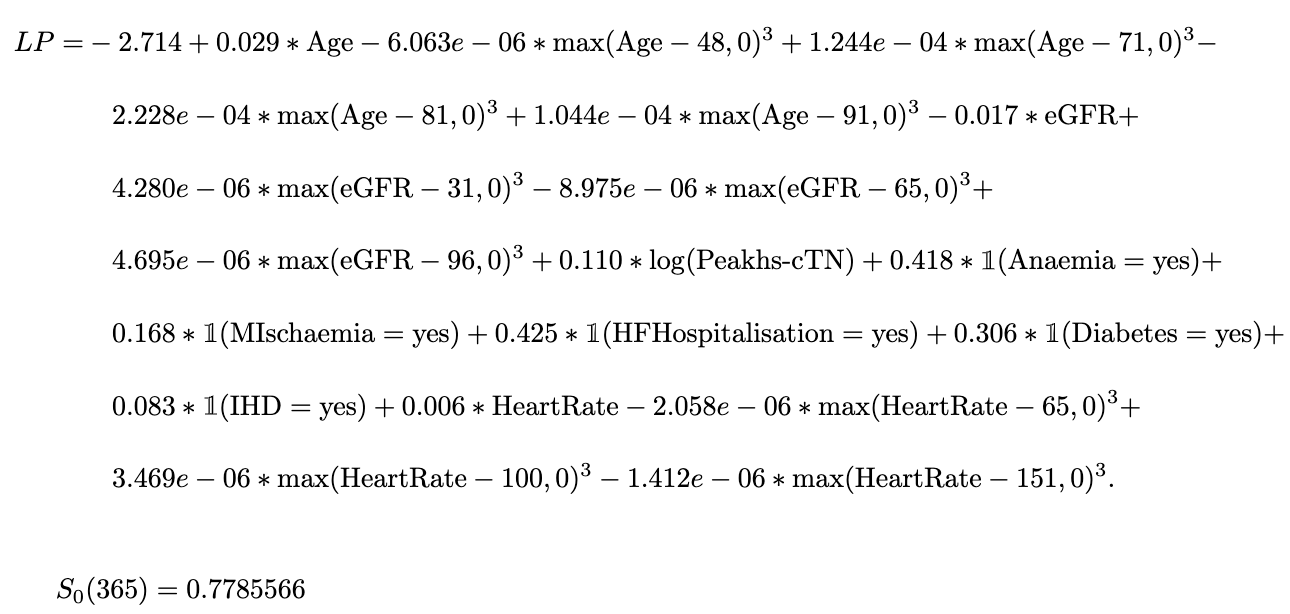


**Supplementary Table 1** –Definition of the ICD-10 codes in single centre validation cohort used to define cardiovascular death.

|  | **Definition** |
| --- | --- |
| **ICD-10 Code** |  |
| I10 | Essential (primary) hypertension |
| I11 | Hypertensive heart disease |
| I12 | Hypertensive chronic kidney disease |
| I13 | Hypertensive heart and chronic kidney disease |
| I15 | Secondary hypertension |
| I20 | Angina pectoris |
| I21 | Acute myocardial infarction |
| I22 | Subsequent ST elevation (STEMI) and non-ST elevation (NSTEMI) myocardial infarction |
| I23 | Certain current complications following ST elevation (STEMI) and non-ST elevation (NSTEMI) myocardial infarction (within the 28 day period) |
| I24 | Other acute ischemic heart diseases |
| I25 | Chronic ischemic heart disease |
| I44 | Atrioventricular and left bundle-branch block |
| I45 | Other conduction disorders |
| I46 | Cardiac arrest |
| I47 | Paroxysmal tachycardia |
| I48 | Atrial fibrillation and flutter |
| I49 | Other cardiac arrhythmias |
| I50 | Heart failure |
| I51 | Complications and ill-defined descriptions of heart disease |
| I61 | Nontraumatic intracerebral haemorrhage |
| I62 | Other and unspecified nontraumatic intracranial haemorrhage |
| I62.9 | Nontraumatic intracerebral haemorrhage, unspecified |
| I63 | Cerebral infarction (all subtypes I63.0-I63.5, I63.8 and I63.9) |
| I64 | Acute cerebrovascular disease |
| I65 | Occlusion and stenosis of precerebral arteries, not resulting in cerebral infarction |
| I66 | Occlusion and stenosis of cerebral arteries, not resulting in cerebral infarction |
| I67 | Other cerebrovascular diseases |
| I70 | Atherosclerosis |
| I71 | Aortic aneurysm and dissection |
| I72 | Other aneurysm |
| I73 | Other peripheral vascular diseases |

**Supplementary Table 2 – Table describing proportions with myocardial injury and infarction in each cohort**

|  | **Derivation cohort**  **(48,282)** | **Single-centre validation cohort**  **(22,589)** | **Multi-centre validation cohort***  **(6,684)** | |
| --- | --- | --- | --- | --- |
| **Cardiac troponin concentration** |  |  |  | |
| >99^th^ Centile URL (%) | 10,360 (21) | 3,853 (17) | 2,529 (38) | |
| <99^th^ Centile URL (%) | 37,922 (79) | 18,736 (83) | 4,119 (61) | |
| **Subtype of myocardial infarction** |  |  |  | |
| Type 1 myocardial infarction (%) | 4,981 (10) | 1,080 (5) | 1,167 (17) | |
| Type 2 myocardial infarction (%) | 1,121 (2) | 250 (1) | 253 (4) | |
| All (%) are given as a total of the complete recruited population  *Troponin results were not available for 39 patients in the multi-centre cohort | | | |  |

**Supplementary Table 3: Baseline characteristics of the derivation cohort stratified by primary outcome**

|  |  | **Derivation cohort** |  | |
| --- | --- | --- | --- | --- |
|  | **Overall**  (n=1,121) | **Primary outcome**  (n=297) | **Censored**  (n=824) | |
| **Demographics** |  |  |  | |
| Age (years), mean (SD) | 74 (14) | 80 (11) | 72 (14) | |
| Male, n (%) | 501 (45%) | 130 (44%) | 371 (45%) | |
| **Past medical history, n (%)** |  |  |  | |
| Myocardial infarction | 163 (15%) | 68 (23%) | 95 (12%) | |
| Ischaemic heart disease ^a^ | 454 (40%) | 159 (54%) | 295 (36%) | |
| Cerebrovascular disease | 135 (12%) | 53 (18%) | 82 (10%) | |
| Diabetes mellitus | 147 (13%) | 60 (20%) | 87 (11%) | |
| Heart failure hospitalisation | 292 (26%) | 121 (41%) | 171 (21%) | |
| Anaemia | 398 (36%) | 156 (54%) | 242 (30%) | |
| **Electrocardiogram, n (%)** |  |  |  | |
| Myocardial ischaemia | 383 (37%) | 113 (42%) | 270 (35%) | |
| **Physiological parameters** |  |  |  | |
| Heart rate (bpm) | 105 (35) | 99 (30) | 107 (37) | |
| Systolic blood pressure (mmHg) | 132 (30) | 130 (30) | 133 (30) | |
| **Haematology and clinical chemistry ^b^** |  |  |  | |
| Haemoglobin | 126 (29) | 117 (29) | 129 (29) | |
| eGFR (mL/min) | 64 (25) | 56 (27) | 66 (24) | |
| Peak hs-cTnI (ng/L) | 125 [48, 604] | 148 [60, 822] | 112 [45, 522] | |
| eGFR calculated according to the MDRD equation (mL/min. ^a^ Defined as prior angina, myocardial infarction, or revascularization.  ^b^Mean (SD), Median [IQR] | | | |  |

**Supplementary Table 4: Comparison of discrimination the GRACE 2.0 score in the single center and multi center external validation cohorts**

| **Validation Cohort** | ***T2-risk***  **AUC, [95%CI]** | **GRACE 2.0**  **AUC, [95%CI]** |
| --- | --- | --- |
| Single-center unselected patient cohort | 82.7, (77.0 – 88.3) | 74.0, (66.8 - 81.1) |
| Multi-center selected patient cohort | 73.8, (64.3 – 83.2) | 71.9, (61.4 – 82.4) |

**Supplementary Table 5: Predictors and characteristics of the primary outcome in the derivation cohort stratified by risk groups.**

|  | | **Derivation cohort** | | | | | | | | |
| --- | --- | --- | --- | --- | --- | --- | --- | --- | --- | --- |
|  | | **Overall**  (n=1,119) | **High risk**  (n=286) | | | **Intermediate risk**  (n=566) | | **Low risk**  (n=267) | | |
| **Demographics** | |  |  | | |  | |  | | |
| Age (years), mean (SD) | | 74 (14) | 84 (9) | | | 76 (10) | | 59 (14) | | |
| Male, n (%) | | 499 (45%) | 137 (48%) | | | 233 (41%) | | 129 (48%) | | |
| **Past medical history, n (%)** | |  |  | | |  | |  | | |
| Ischaemic heart disease ^a^ | | 453 (40%) | 182 (64%) | | | 229 (40%) | | 42 (16%) | | |
| Cerebrovascular disease | | 134 (12%) | 50 (17%) | | | 72 (10%) | | 12 (4.5%) | | |
| Diabetes mellitus | | 146 (13%) | 81 (28%) | | | 58 (10%) | | 7 (2.6%) | | |
| Heart failure hospitalisation | | 291 (26%) | 168 (59%) | | | 113 (20%) | | 10 (3.7%) | | |
| Anaemia | | 406 (36%) | 208 (73%) | | | 174 (31%) | | 24 (9.0%) | | |
| **Electrocardiogram, n (%)** | |  |  | | |  | |  | | |
| Myocardial ischaemia | | 417 (37%) | 134 (47%) | | | 211 (37%) | | 72 (27%) | | |
| **Physiological parameters** | |  |  | | |  | |  | | |
| Heart rate (bpm) | | 105 (35) | 94 (22) | | | 102 (32) | | 122 (45%) | | |
| Systolic blood pressure (mmHg) | | 132 (30) | - | | | - | | - | | |
| **Hematology and clinical chemistry ^b^** | |  |  | | |  | |  | | |
| Haemoglobin | | 126 (29) | - | | | - | | - | | |
| eGFR (mL/min) | | 64 (25) | 49 (27) | | | 65 (23) | | 77 (19) | | |
| Peak hs-cTnI (ng/L) | | 125 [48, 604] | 148 [60, 822] | | | 115 [48, 457] | | 75 [40, 204] | | |
| **Mechanism of myocardial injury (%)*** |  | |  |  | |  | | |  |  |
| Coronary mechanisms | 35 (3%) | | <5 (<2%) | 16 (2.8%) | | <20 (<7%) | | |  |  |
| Systemic mechanisms | 462 (20%) | | 184 (64%) | 219 (39%) | | 59 (22%) | | |  |  |
| Tachyarrhythmia | 616 (55%) | | 100 (35%) | 327 (58%) | | 189 (71%) | | |  |  |
| eGFR calculated according to the MDRD equation (mL/min. ^a^ Defined as prior angina, myocardial infarction, or revascularization.  ^b^Mean (SD), Median [IQR]. There were 6 patients for which the mechanisms of myocardial injury cloud not be determined. | | | | | | | | |  |  |

**Supplementary Table S6: Sensitivity analysis of imputed versus complete case analysis**

|  | **Imputed data**  **n = 1,119**  **AUC (95%CI)** | **Unimputed data**  **n = 817**  **AUC (95%CI)** | **DeLong test**  **(p value)** |
| --- | --- | --- | --- |
| **T2-risk**  **Primary outcome** | 0.756 95% CI (0.73-0.79) | 0.769 95% CI (0.73-0.81) | 0.59 |
| **T2-risk**  **Secondary outcome** | 0.745 95% CI (0.70-0.79) | 0.753 95% CI (0.70-0.80) | 0.82 |

**Code used to generate models**

The R code used to generate the T2-risk model is available at <https://github.com/KarlaMonterrubioG/T2-risk-score>.

Similarly, an app has been developed and can be found at

<https://t2score.shinyapps.io/t2_riskscore/>

**References**

1. Thygesen K, Alpert JS, Jaffe AS, et al. Fourth universal definition of myocardial infarction (2018). Eur. Heart J. 2019.

2. Thygesen K, Mair J, Giannitsis E, et al. How to use high-sensitivity cardiac troponins in acute cardiac care. Eur. Heart J. 2012;33:2252–7.

3. Apple FS, Jesse RL, Newby LK, Wu AHB, Christenson RH. National Academy of Clinical Biochemistry and IFCC Committee for Standardization of Markers of Cardiac Damage Laboratory Medicine Practice Guidelines: Analytical issues for biochemical markers of acute coronary syndromes. Circulation 2007;115:e352-5.

4. Collet J-P, Thiele H, Barbato E, et al. 2020 ESC Guidelines for the management of acute coronary syndromes in patients presenting without persistent ST-segment elevation: The Task Force for the management of acute coronary syndromes in patients presenting without persistent ST-segment elevation. Eur. Heart J. 2020.

**TRIPOD statement**

| **Section/Topic** | **Item** |  | **Checklist Item** | **Page** |
| --- | --- | --- | --- | --- |
| **Title and abstract** | | | | |
| Title | 1 | D;V | Identify the study as developing and/or validating a multivariable prediction model, the target population, and the outcome to be predicted. | 1 |
| Abstract | 2 | D;V | Provide a summary of objectives, study design, setting, participants, sample size, predictors, outcome, statistical analysis, results, and conclusions. | 4-5 |
| **Introduction** | | | | |
| Background and objectives | 3a | D;V | Explain the medical context (including whether diagnostic or prognostic) and rationale for developing or validating the multivariable prediction model, including references to existing models. | 8 |
|  | 3b | D;V | Specify the objectives, including whether the study describes the development or validation of the model or both. | 8 |
| **Methods** | | | | |
| Source of data | 4a | D;V | Describe the study design or source of data (e.g., randomized trial, cohort, or registry data), separately for the development and validation data sets, if applicable. | 9-10 |
|  | 4b | D;V | Specify the key study dates, including start of accrual; end of accrual; and, if applicable, end of follow-up. | 9-10 |
| Participants | 5a | D;V | Specify key elements of the study setting (e.g., primary care, secondary care, general population) including number and location of centres. | 9-10 |
|  | 5b | D;V | Describe eligibility criteria for participants. | 9-10 |
|  | 5c | D;V | Give details of treatments received, if relevant. | NA |
| Outcome | 6a | D;V | Clearly define the outcome that is predicted by the prediction model, including how and when assessed. | 11-12 |
|  | 6b | D;V | Report any actions to blind assessment of the outcome to be predicted. | 10 +  Supplementary appendix 2-4 |
| Predictors | 7a | D;V | Clearly define all predictors used in developing or validating the multivariable prediction model, including how and when they were measured. | 13-14 |
|  | 7b | D;V | Report any actions to blind assessment of predictors for the outcome and other predictors. | NA |
| Sample size | 8 | D;V | Explain how the study size was arrived at. | 15 + figure 1 |
| Missing data | 9 | D;V | Describe how missing data were handled (e.g., complete-case analysis, single imputation, multiple imputation) with details of any imputation method. | Supplementary code 1.3 and 4.1 |
| Statistical analysis methods | 10a | D | Describe how predictors were handled in the analyses. | 13-14 |
|  | 10b | D | Specify type of model, all model-building procedures (including any predictor selection), and method for internal validation. | 13-14 |
|  | 10c | V | For validation, describe how the predictions were calculated. | Supplementary code: External validation |
|  | 10d | D;V | Specify all measures used to assess model performance and, if relevant, to compare multiple models. | 13-14  Supplementary code: External validation |
|  | 10e | V | Describe any model updating (e.g., recalibration) arising from the validation, if done. | 13 |
| Risk groups | 11 | D;V | Provide details on how risk groups were created, if done. | 13-14 |
| Development vs. validation | 12 | V | For validation, identify any differences from the development data in setting, eligibility criteria, outcome, and predictors. | 9-10 |
| **Results** | | | | |
| Participants | 13a | D;V | Describe the flow of participants through the study, including the number of participants with and without the outcome and, if applicable, a summary of the follow-up time. A diagram may be helpful. | Figure 1 |
|  | 13b | D;V | Describe the characteristics of the participants (basic demographics, clinical features, available predictors), including the number of participants with missing data for predictors and outcome. | Table 1  Table 2  Supplement S1 |
|  | 13c | V | For validation, show a comparison with the development data of the distribution of important variables (demographics, predictors and outcome). | 9-10  Table1  Table 2 |
| Model development | 14a | D | Specify the number of participants and outcome events in each analysis. | 16-17  Table 2 |
|  | 14b | D | If done, report the unadjusted association between each candidate predictor and outcome. | Table 3  Supplementary code 2.1 |
| Model specification | 15a | D | Present the full prediction model to allow predictions for individuals (i.e., all regression coefficients, and model intercept or baseline survival at a given time point). | Supplementary code: 2.2 |
|  | 15b | D | Explain how to the use the prediction model. | Supplementary Code 2.2  Shiny App link page 14 manuscript and 15 supplement |
| Model performance | 16 | D;V | Report performance measures (with CIs) for the prediction model. | Table 3  Supplementary code section 2 |
| Model-updating | 17 | V | If done, report the results from any model updating (i.e., model specification, model performance). | NA |
| **Discussion** | | | | |
| Limitations | 18 | D;V | Discuss any limitations of the study (such as nonrepresentative sample, few events per predictor, missing data). | 20-21 |
| Interpretation | 19a | V | For validation, discuss the results with reference to performance in the development data, and any other validation data. | 19-21 |
|  | 19b | D;V | Give an overall interpretation of the results, considering objectives, limitations, results from similar studies, and other relevant evidence. | 19-21 |
| Implications | 20 | D;V | Discuss the potential clinical use of the model and implications for future research. | 4-5, 7 |
| **Other information** | | | | |
| Supplementary information | 21 | D;V | Provide information about the availability of supplementary resources, such as study protocol, Web calculator, and data sets. | 11 ,17 and in supplement 15 – shiny app link |
| Funding | 22 | D;V | Give the source of funding and the role of the funders for the present study. | 2 |

*Items relevant only to the development of a prediction model are denoted by D, items relating solely to a validation of a prediction model are denoted by V, and items relating to both are denoted D;V. We recommend using the TRIPOD Checklist in conjunction with the TRIPOD Explanation and Elaboration document
